# TRIM16 facilitates SIRT‐1‐dependent regulation of antioxidant response to alleviate age‐related sarcopenia

**DOI:** 10.1002/jcsm.13553

**Published:** 2024-08-27

**Authors:** Ai Guo, Ke Huang, Quanyi Lu, Bailong Tao, Kai Li, Dianming Jiang

**Affiliations:** ^1^ Department of Orthopedics The Third Affiliated Hospital of Chongqing Medical University Chongqing China; ^2^ Department of Critical Care Medicine The First Affiliated Hospital of Chongqing Medical University Chongqing China; ^3^ Laboratory Research Center The First Affiliated Hospital of Chongqing Medical University Chongqing China

**Keywords:** Muscle atrophy, Oxidative stress, Sarcopenia, TRIM16

## Abstract

**Background:**

Age‐related sarcopenia, characterized by reduced skeletal muscle mass and function, significantly affects the health of the elderly individuals. Oxidative stress plays a crucial role in the development of sarcopenia. Tripartite motif containing 16 (TRIM16) is implicated in orchestrating antioxidant responses to mitigate oxidative stress, yet its regulatory role in skeletal muscle remains unclear. This study aims to elucidate the impact of TRIM16 on enhancing antioxidant response through SIRT‐1, consequently mitigating age‐related oxidative stress, and ameliorating muscle atrophy.

**Methods:**

Aged mouse models were established utilizing male mice at 18 months with D‐galactose (D‐gal, 200 mg/kg) intervention and at 24 months with natural aging, while 3‐month‐old young mice served as controls. Muscle cell senescence was induced in C2C12 myoblasts using 30 g/L D‐gal. TRIM16 was overexpressed in the skeletal muscle of aged mice and silenced/overexpressed in C2C12 myoblasts. The effects of TRIM16 on skeletal muscle mass, grip strength, morphological changes, myotube formation, myogenic differentiation, and muscle atrophy indicators were evaluated. Reactive oxygen species (ROS) levels and oxidative stress‐related parameters were measured. The SIRT‐1 inhibitor EX‐527 was employed to elucidate the protective role of TRIM16 mediated through SIRT‐1.

**Results:**

Aged mice displayed significant reductions in lean mass (−11.58%; −14.47% vs. young, *P* < 0.05), hindlimb lean mass (−17.38%; −15.95% vs. young, *P* < 0.05), and grip strength (−22.29%; −31.45% vs. young, *P* < 0.01). Skeletal muscle fibre cross‐sectional area (CSA) decreased (−29.30%; −24.12% vs. young, *P* < 0.05). TRIM16 expression significantly decreased in aging skeletal muscle (−56.82%; −66.27% vs. young, *P* < 0.001) and senescent muscle cells (−46.53% vs. control, *P* < 0.001). ROS levels increased (+69.83% vs. control, *P* < 0.001), and myotube formation decreased in senescent muscle cells (−56.68% vs. control, *P* < 0.001). Expression of myogenic differentiation and antioxidant indicators decreased, while muscle atrophy markers increased in vivo and in vitro (all *P* < 0.05). Silencing TRIM16 in myoblasts induced oxidative stress and myotube atrophy, while TRIM16 overexpression partially mitigated aging effects on skeletal muscle. TRIM16 activation enhanced SIRT‐1 expression (+75.38% vs. control, *P* < 0.001). SIRT‐1 inhibitor EX‐527 (100 μM) suppressed TRIM16's antioxidant response and mitigating muscle atrophy, offsetting the protective effect of TRIM16 on senescent muscle cells.

**Conclusions:**

This study elucidates TRIM16's role in mitigating oxidative stress and ameliorating muscle atrophy through the activation of SIRT‐1‐dependent antioxidant effects. TRIM16 emerges as a potential therapeutic target for age‐related sarcopenia.

## Introduction

Sarcopenia, a geriatric syndrome characterized by accelerated loss of muscle mass and function, poses a significant risk for physical frailty, disability, and mortality among the elderly.^1^ Recent studies have elucidated numerous potential risk factors associated with sarcopenia, including alterations in body composition, hormonal changes, muscle denervation, and diminished regenerative capacity of satellite cells. Additionally, mitochondrial dysfunction, oxidative stress, and chronic inflammatory responses are recognized as crucial molecular mechanisms contributing to the onset and progression of sarcopenia.^2^, ^3^

Oxidative stress is a central mechanism in the pathogenesis of sarcopenia. Excessive ROS production in aged skeletal muscle disrupts the redox balance, leading to oxidative stress and ultimately contributing to the onset and progression of sarcopenia.^4^, ^5^, ^6^ Elevated ROS levels in aged skeletal muscle can hinder the Akt/mTOR signalling pathway, reducing muscle protein synthesis, and upregulate FOXOs signalling, increasing the expression of muscle‐specific E3 ubiquitin ligases such as muscle atrophy F‐box (MAFbx)/Atrogin‐1 and muscle RING finger 1 (MuRF1).^7^ Oxidative stress also inhibits satellite cell proliferation and differentiation, further disrupting muscle homeostasis.^8^ Therefore, investigating key proteins involved in oxidative stress offers a promising avenue for identifying new therapeutic targets for age‐related sarcopenia.

Tripartite motif containing 16 (TRIM16), an E3 ubiquitin ligase belonging to the TRIM protein family, exhibits widespread expression across various tissues and cell types.^9^ Its role as an inhibitor of tumour cell growth and metastasis positions TRIM16 as a potential therapeutic target for cancer diseases.^10^ Studies indicate that TRIM16 functions as a scaffold protein, interacting with autophagy‐related proteins like P62, ULK1, ATG16L1, and LC‐3, to facilitate autophagosome biogenesis.^11^, ^12^ Additionally, TRIM16 works with Galectin‐3 to maintain lysosomal autophagic homeostasis by selectively sequestering damaged lysosomes, thus protecting cells from lysosomal damage.^13^ TRIM16 has emerged as a novel regulatory protein responsive to oxidative stress, enhancing cellular defences against oxidative damage by bolstering antioxidant systems.^14^ Typically suppressed by cytoplasmic Keap1, Nuclear factor erythroid 2‐related factor 2 (Nrf‐2) translocates to the nucleus upon exposure to ROS, facilitating the transcription of antioxidant proteins.^15^ TRIM16 stabilizes and activates Nrf‐2, aiding in the antioxidant response.^16^ Studies show that TRIM16 protects human periodontal ligament stem cells (hPDLSCs) from oxidative stress by activating the PICOT/Akt/Nrf‐2 pathway, preserving osteogenic differentiation potential.^17^ In HEK293T cells, TRIM16 knockdown increases apoptosis under oxidative stress, while its overexpression elevates antioxidant factors like NQO1 and HO‐1.^11^ However, TRIM16's role and mechanism in skeletal muscle aging remain unclear and require further investigation.

Silent information regulator 1 (SIRT‐1), an NAD‐dependent histone deacetylase, plays a key role with antioxidant, anti‐inflammatory, and anti‐apoptotic properties.^18^ SIRT‐1 activity decreases with age, leading to higher ROS production.^19^ Increased SIRT‐1 levels are associated with nuclear accumulation of Nrf‐2 and enhanced Nrf‐2‐mediated gene expression.^20^ SIRT‐1 boosts antioxidant effects by activating PGC‐1α and increasing the expression of enzymes like superoxide dismutase (SOD), catalase, and glutathione peroxidase 1 (GPx1).^21^ It also deacetylates and activates endothelial nitric oxide synthase (eNOS), raising nitric oxide (NO) production.^22^ This highlights the protective effect of SIRT1 against oxidative stress through antioxidant mechanisms. However, the role of TRIM16 in facilitating SIRT‐1 against sarcopenia requires further investigation.

The study delved into the role of TRIM16 in initiating an antioxidant response via SIRT‐1, both in vitro and in vivo. Our objective was to evaluate whether this mechanism could effectively mitigate oxidative stress in aging skeletal muscle, thereby attenuating muscle atrophy. These findings may present a promising therapeutic target for sarcopenia treatment.

## Materials and methods

### Animals and treatment

Male C57BL/6J mice, aged 4–5 weeks and 11–12 months, were obtained from and housed at the Experimental Animal Center of Chongqing Medical University, P. R. China, following ethical guidelines. Animal experiments followed the principles in the Guide for the Care and Use of Laboratory Animals by the National Institutes of Health and were approved by the Animal Ethics Committee of Chongqing Medical University. After acclimatization, mice were randomly assigned to four groups based on their exposure to D‐galactose (D‐gal) solution: (1) 3‐month‐old young control group (young group, *n* = 6); (2) 18‐month‐old control group (18‐month‐old group, *n* = 6); (3) 18‐month‐old‐D‐gal group (18‐month‐D‐gal group, *n* = 12); (4) 24‐month‐old control group (24‐month‐old group, *n* = 12). Mice in the 18‐month‐D‐gal group were intraperitoneally injected with D‐gal solution (200 mg/kg) for 8 weeks, while the 18‐month‐old group received saline as the control. Researchers collecting data in this study were not blinded to the experimental manipulations performed on each experimental animal.

Mice from both the 18‐month‐D‐gal group and the 24‐month‐old group were randomly divided into four subgroups for adeno‐associated virus 9 (AAV9) administration: (1) 18‐month‐D‐gal+AAV‐Control group (18‐month‐D‐gal+AAV‐Con group, *n* = 6); (2) 18‐month‐D‐gal+AAV‐TRIM16‐overexpression group (18‐month‐D‐gal+AAV‐TRIM16 group, *n* = 6); (3) 24‐month‐old+AAV‐Control group (24‐month‐old+AAV‐Con group, *n* = 6); (4) 24‐month‐old+AAV‐TRIM16‐overexpression group (24‐month‐old+AAV‐TRIM16 group, *n* = 6). AAV serotype 9 vectors (pHBAAV‐CMV‐MCS‐3flag‐T2A‐ZsGreen) were obtained from Hanheng Biotechnology Co., Ltd. (Shanghai, China). For AAV9 administration, the hindlimbs of the mice were shaved, and each gastrocnemius muscle received total 50 μL of 10^12^ vg/mL AAV‐Con or AAV‐TRIM16 virus (3 sites/side, 10–20 μL/site). Transfection efficiency was assessed 6 weeks after AAV9 injection.

### Cell culture and treatment

The C2C12 cell line (Shanghai Zhong Qiao Xin Zhou Biotechnology, China) was cultured in complete DMEM culture medium supplemented with 10% fetal bovine serum and 1% penicillin–streptomycin. Upon reaching 80–90% confluence, cells were switched to differentiation medium consisting of 2% horse serum and 1% penicillin–streptomycin to facilitate myotube formation. Cells were treated with differentiation medium containing various concentrations of D‐gal (0, 10, 20, 30, and 40 g/L, Sigma, USA) for 48 h to induce cellular senescence. In specific experiments, cells were treated with a SIRT‐1 inhibitor (EX‐527, MCE, USA) for 48 h. Lentiviral plasmids encoding TRIM16 and a negative control were synthesized and constructed from Hanheng Biotechnology Co., Ltd. (Shanghai, China). C2C12 cells were transfected with the lentivirus (pHBLV‐U6‐MCS‐EF1‐mcherry‐T2A‐PURO & pHBLV‐CMV‐MCS‐3flag‐EF1‐mCherry‐T2A‐puro) following the manufacturer's instructions. Positive cells were selected with puromycin (Beyotime Biotechnology, China), and TRIM16 knockdown or overexpression efficiency was evaluated via immunoblotting. The sequences of synthesized shRNA targeting TRIM16 and the control scrambled shRNA are provided in Table S1.

### Dual‐energy X‐ray absorptiometry

All experimental mice were anaesthetised and placed prone on the scanner bed for body composition analysis using dual‐energy X‐ray absorptiometry (DEXA; Hologic Discovery A [Hologic Inc., USA]).

### Grip strength test

The grip strength test was conducted using an electronic grip strength meter (Cat. 47200, Ugo Basile). The mouse's forelimbs were placed flat on the sensing rod of the grip strength meter. After confirming the mouse's grip, its tail was gently pulled backward in a parallel direction until the mouse released its grip. The grip strength meter automatically recorded the maximum grip force of each mouse, with three repeated tests conducted.

### Tissue preparation

After weighing the mice, anaesthesia was administered via intraperitoneal injection of 3% pentobarbital sodium (0.1–0.2 mL/10 g). Gastrocnemius (GAS) muscles were promptly harvested, rapidly frozen in liquid nitrogen, and stored at −80°C for subsequent western blot analysis. Furthermore, a subset of GAS muscles was fixed in 4% paraformaldehyde at 4°C overnight. After fixation, these muscles were embedded in paraffin and sliced sequentially at a thickness of 4–6 μm for further histological analysis.

### Transmission electron microscope

The GAS muscle samples were immediately fixed in 2.5% glutaraldehyde and sectioned into multiple blocks measuring 1–3 mm in size. From these blocks, a random subset was selected for subsequent transmission electron microscopy (TEM) experiments. The chosen blocks underwent processing and screening for TEM analysis (Hitachi, Japan).

### Haematoxylin and eosin staining and immunohistochemistry

The tissue sections were dewaxed and hydrated, followed by haematoxylin staining for 3 min and immersion in eosin for 2 min. After dehydration and clearing, slides were mounted using neutral resin. Subsequently, sections were examined under a microscope, and digital images were captured with a camera. For immunohistochemical (IHC) analysis, tissue sections underwent deparaffinization and hydration. Heat‐induced epitope retrieval (HIER) was performed, followed by treatment with 3% H_2_O_2_ for 20 min and exposure to 10% goat serum for 30 min at 37°C. Next, sections were incubated overnight at 4°C with the primary antibody, followed by incubation with the secondary antibody IgG‐HRP for 30 min. Immunoreactivity was visualized using 3,3′‐diaminobenzidine tetrahydrochloride (DAB). Finally, sections were dehydrated and mounted on slides. All sections were examined under a light microscope (ZEISS, Germany), and images were analysed using ImageJ software.

### Immunofluorescence

C2C12 cells cultured in 6‐well plates were fixed with 4% paraformaldehyde (PFA) for 20 min and permeabilized with 0.3% Triton‐X for 10 min. Additionally, GAS muscles stored at −80°C were immediately sliced into 10 μm sections and mounted onto slides. The C2C12 cells or frozen muscle sections were washed with PBS and blocked with 10% normal goat serum for 1 h. Subsequently, the samples were incubated with the primary antibodies (see Table S2) at 4°C overnight. After washing, the samples underwent incubation with a fluorescent secondary antibody for 1 h. Nuclei were stained with DAPI for 8 min. Images were acquired using laser confocal scanning microscopy (LCSM, Zeiss).

### Senescence‐associated β‐galactosidase staining

After treatment, cells were subjected to senescence‐associated β‐galactosidase (SA‐β‐gal) staining using the SA‐β‐gal staining kit (Beyotime Biotechnology, China). Following staining, cells were cultured in a 37°C dry incubator without CO_2_. SA‐β‐gal positive cells were visualized under a light microscope.

### Intracellular reactive oxygen species level

Intracellular reactive oxygen species (ROS) levels were assessed using a ROS Assay Kit (Beyotime Biotechnology, China). Following the intervention, cells in confocal dishes were treated with 2′,7′‐dichlorofluorescein diacetate (DCFH‐DA) dissolved in DMEM and incubated at 37°C for 30 min.

### Detection of glutathione, malondialdehyde contents, and activity of superoxide dismutase

Glutathione (GSH) and malondialdehyde (MDA) levels were quantified using the GSH assay kit (Solarbio, China) and MDA assay kit (Beyotime Biotechnology, China), respectively, following the manufacturer's protocols. Superoxide dismutase (SOD) activity was assessed using the SOD assay kit (Beyotime Biotechnology, China).

### Cell apoptosis measurement

C2C12 cells were harvested using 0.25% pancreatic enzyme and washed three times with PBS buffer. The cell suspension was transferred to an EP tube, and the processed samples were analysed using flow cytometry.

### Western blotting analysis

C2C12 cells and GAS muscles were lysed using RIPA buffer (Beyotime Biotechnology, China) supplemented with PMSF (1:100) and phosphatase inhibitor (1:50). Equal amounts of protein (30 μg) were separated by 10% SDS‐PAGE, transferred onto PVDF membranes (Millipore, USA), and blocked with a milk solution for 2 h at room temperature. The membranes were then incubated overnight at 4°C with primary antibodies (see Table S2). After washing, the membranes were incubated with secondary antibodies for 1 h. Protein bands were visualized using an enhanced chemiluminescence reagent (Advansta, USA), and the bands were analysed using the Fusion imaging system (Fusion Imaging Software, USA).

### Statistical analysis

All experiments were performed independently at least three times. Results were presented as mean ± standard deviation (SD) and analysed using GraphPad Prism 10 software. The Shapiro–Wilk test (*P* > 0.05) and Q–Q plots were employed to analyse the normality of the pooled data distribution. Student's *t*‐test was employed for comparisons between two groups, while one‐way ANOVA (Tukey's test or Kruskal–Wallis test) was used for multiple comparisons involving three or more groups. Statistical significance was considered at a *P*‐value less than 0.05 (*P* < 0.05).

## Results

### D‐galactose‐induced senescence in C2C12 muscle cells

**Figure 1:**
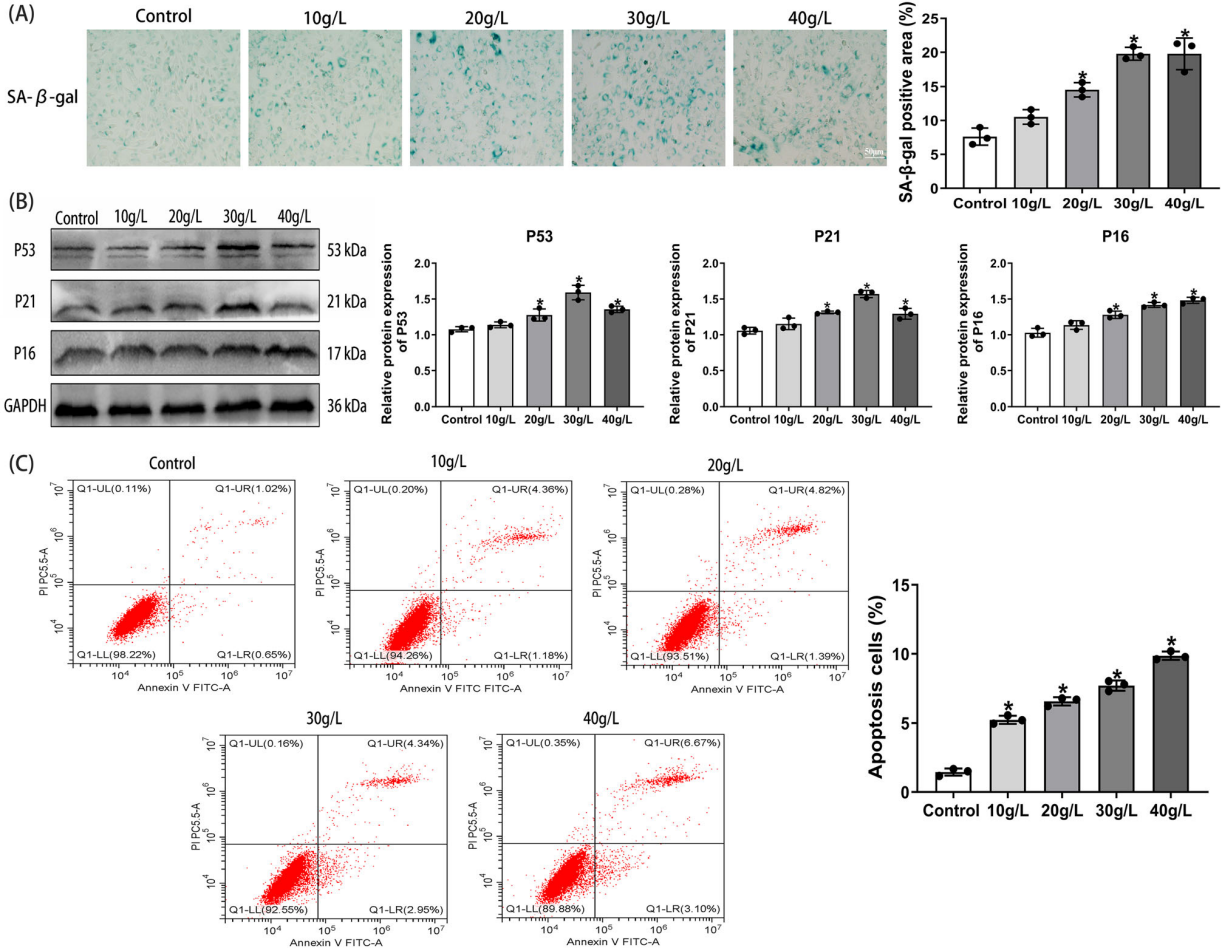
D‐galactose‐induced senescence in C2C12 muscle cells. (A) Visualization of senescent cells through SA‐β‐gal staining (scale bar = 50 μm). (B) Western blot analysis depicting protein expression levels of aging markers P53, P21, and P16. (C) Flow cytometry analysis revealing percentages of apoptotic cells. All data are presented as mean ± SD, *n* = 3. *P < 0.05 compared with the control group.

In previous studies, it has been demonstrated that D‐galactose (D‐gal) can induce senescence in C2C12 cells.^23^, ^24^ In this study, we utilized varying concentrations of D‐gal (0, 10, 20, 30, and 40 g/L) to establish aging muscle cell models. The Cell Counting Kit‐8 (CCK8) results revealed a significant decrease in the proliferation activity of C2C12 cells at 24 and 48 h with increasing concentrations of D‐gal intervention (Figure S1a). SA‐β‐gal staining was employed to evaluate C2C12 cell senescence, revealing a gradual increase in the number of SA‐β‐gal‐positive cells, stained blue, as the concentration of D‐gal increased (Figure 1A). Exposure of C2C12 myotubes to D‐gal concentrations ranging from 20 to 40 g/L resulted in a significant upregulation of senescent markers (P53, P21, and P16) (Figure 1B). Muscle cell aging is characterized by an increase in apoptosis, and flow cytometry results indicated a significant increase in the total apoptosis rate of cells following D‐gal intervention (Figure 1C). A decrease in mitochondrial membrane potential, a hallmark event in early apoptosis, was observed in cells stained with JC‐1, displaying a reduction in red fluorescence and an increase in green fluorescence (Figure S1c). Desmin, an intermediate filament protein, plays a fundamental role in maintaining the structural integrity of skeletal muscle cells.^25^ Immunofluorescence analysis of desmin revealed notable cytoskeletal disarray and shrinkage in muscle cells treated with D‐gal (Figure S1b). These findings collectively indicate that D‐gal treatment successfully induces senescence in C2C12 muscle cells.

### Oxidative stress and myotube atrophy in D‐galactose‐induced senescence of C2C12 muscle cells

**Figure 2:**
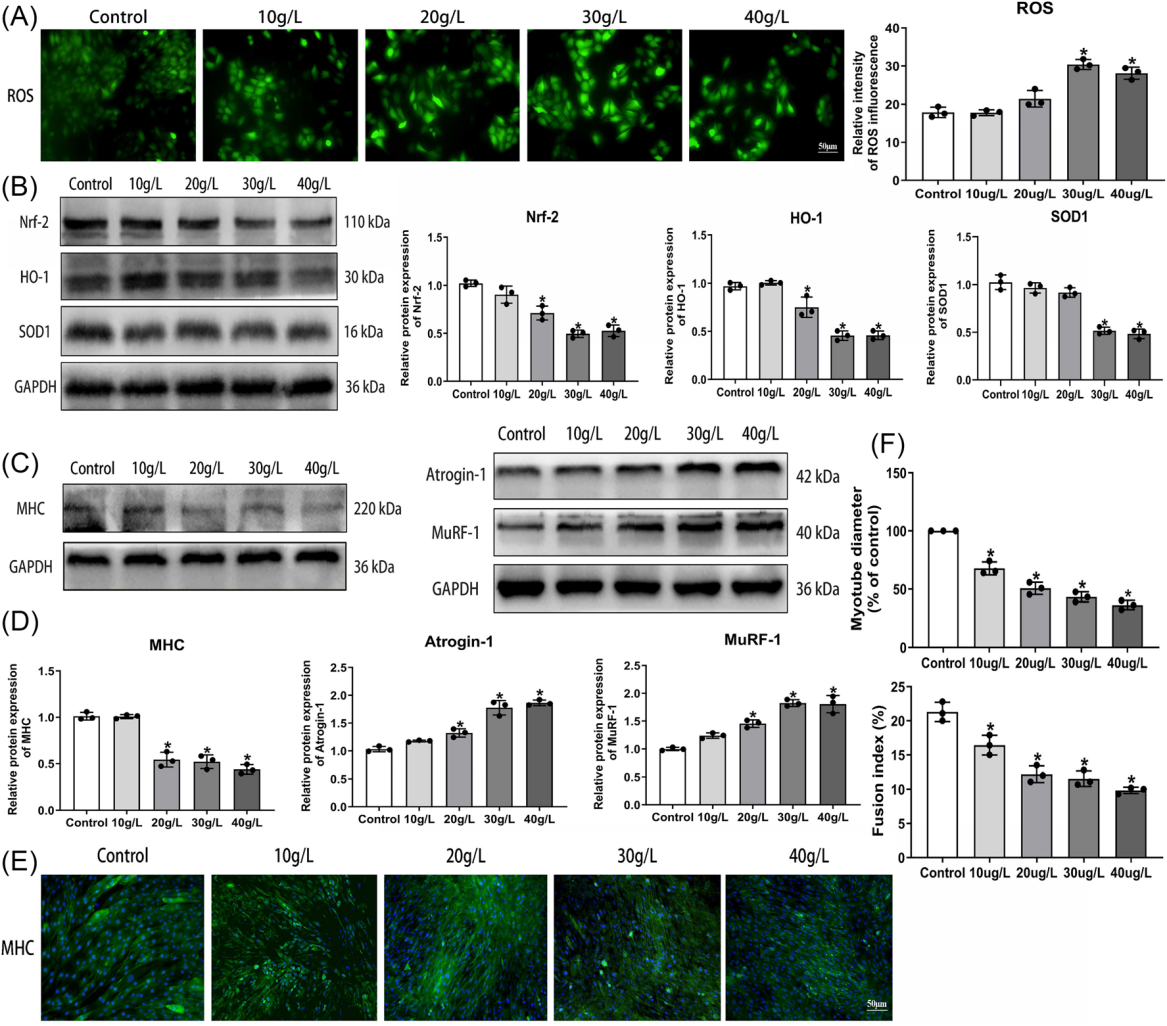
Oxidative stress and myotube atrophy in D‐galactose‐induced senescence of C2C12 muscle cells. (A) Immunofluorescence assessment of ROS levels (scale bar = 50 μm). (B) Western blot analysis depicting protein expression of antioxidant indicators. (C, D) Western blot detection of protein expression related to muscle differentiation and atrophy markers. (E, F) Immunofluorescence detection of myosin heavy chain (MHC) for myotube differentiation (scale bar = 50 μm). All data are presented as mean ± SD, *n* = 3. *P < 0.05 compared with the control group.

Analysis of intracellular ROS levels showed a notable elevation in ROS production in C2C12 cells upon exposure to D‐gal (Figure 2A). Western blot analysis revealed a reduction in the expression of antioxidant factors, including Nrf‐2, HO‐1, and SOD1 following D‐gal stimulation (Figure 2B). Concomitantly, the expression levels of Keap1 and NOX4 increased (Figure S2a). Furthermore, western blotting revealed reduced expression of myogenic differentiation markers (MHC, MyoD, and MyoG) (Figures 2C,D and S2b) and increased expression of muscle atrophy markers (Atrogin‐1 and MuRF‐1) at D‐gal concentrations of 20 to 40 g/L (Figure 2C,D). Immunofluorescent staining of MHC further illustrated a reduction in myotube diameter and fusion index (Figure 2E,F). These findings indicate that D‐gal‐induced senescent muscle cells exacerbate oxidative stress and impede myogenic differentiation leading to myotube atrophy.

### Skeletal muscle atrophy and suppressed antioxidant activity in aged mice

**Figure 3:**
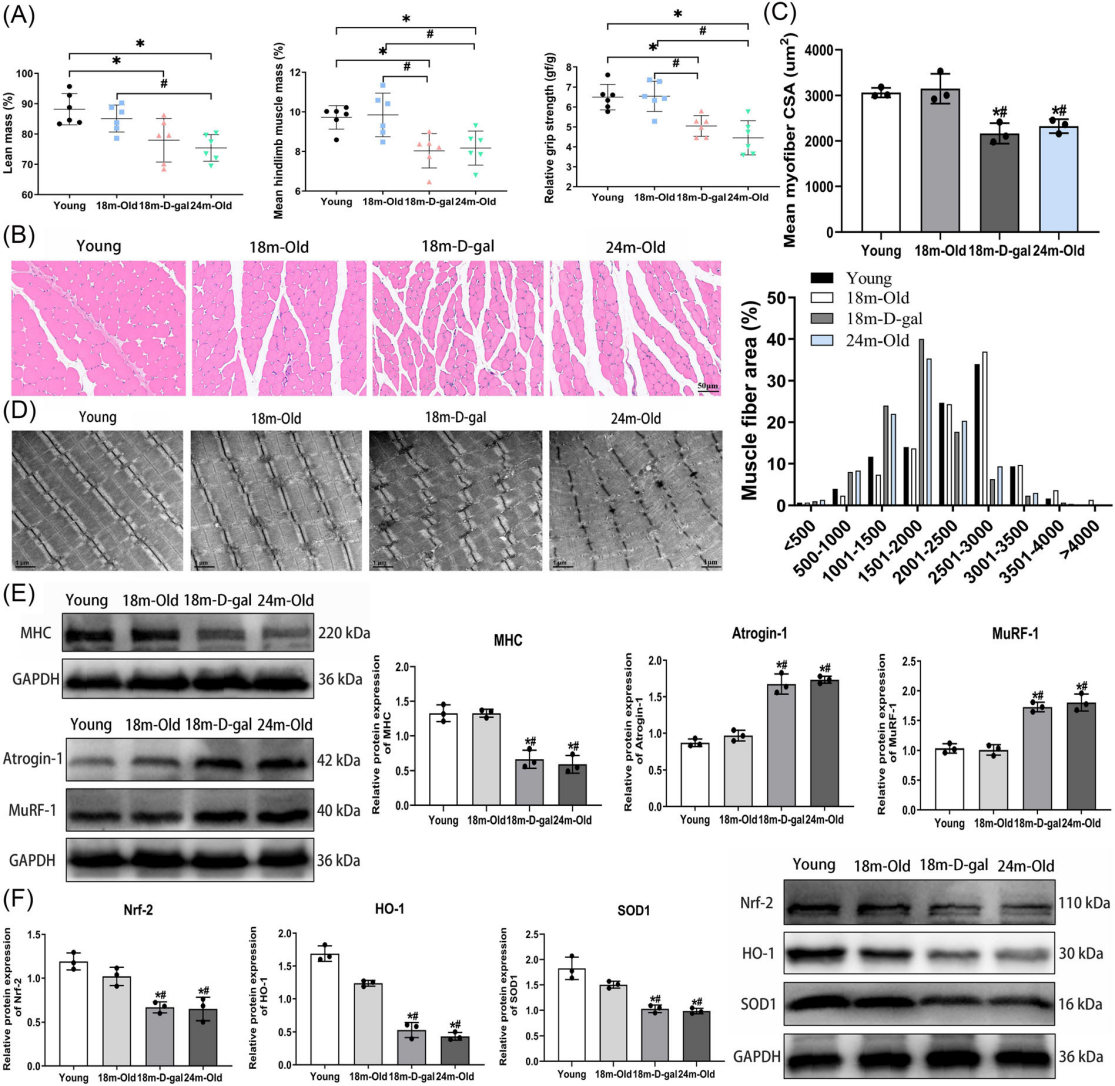
Skeletal muscle atrophy and suppressed antioxidant activity in aged mice. (A) Comparison of lean mass, mean hindlimb muscle mass, and relative grip strength among different groups of mice. (B) HE staining for assessing the morphology of muscle fibres (scale bar = 50 μm). (C) Quantitative analysis of the CSA of muscle fibres in different groups. (D) TEM used to observe the morphology of muscle fibres (scale bar = 1 μm). (E) Western blot analysis of muscle differentiation and muscle atrophy marker protein expression. (F) Western blot analysis of antioxidant indicators protein expression. All data are presented as mean ± SD, *n* = 6. *P < 0.05 compared with the young group, ^#^ *P* < 0.05 compared with the 18‐month‐old group.

In this investigation, two aging mouse models were established, utilizing naturally aged mice and mice subjected to D‐gal intervention. DEXA analysis revealed a significant reduction in lean mass and mean hindlimb mass in both the 24‐month‐old group and 18‐month‐D‐gal group, accompanied by a notable decrease in relative grip strength (Figure 3A). Haematoxylin and eosin (HE) staining and Laminin fluorescence staining of the GAS muscle unveiled a noteworthy reduction in the cross‐sectional area (CSA) of muscle fibres in both the 24‐month‐old group and the 18‐month‐D‐gal group compared with the young group, alongside a leftward shift in fibre distribution (Figures 3B,C, and S3b). Additionally, TEM revealed disturbances in myofibrillar alignment and blurred sarcomeres in the GAS muscles of aged mice (Figure 3D). Western blot analysis showed a significant decrease in myogenic differentiation marker expression (Figures 3E and S3d). In contrast, muscle atrophy markers were significantly increased in the 18‐month‐D‐gal and 24‐month‐old groups compared with the young and 18‐month‐old groups (Figure 3E). However, no statistically significant variances were observed in above parameters between the 24‐month‐old group and 18‐month‐D‐gal group. These findings suggest that aged mice experience muscle atrophy, leading to reduced muscle mass and functionality. Additionally, a decrease in antioxidant factors (Nrf‐2, HO‐1, and SOD1) was observed (Figure 3F), while the expression of Keap1 and NOX4 increased in the 18‐month‐D‐gal and 24‐month‐old groups (Figure S3c).

### Downregulation of TRIM16 in skeletal muscle of aged mice and D‐galactose‐induced senescent muscle cells

**Figure 4:**
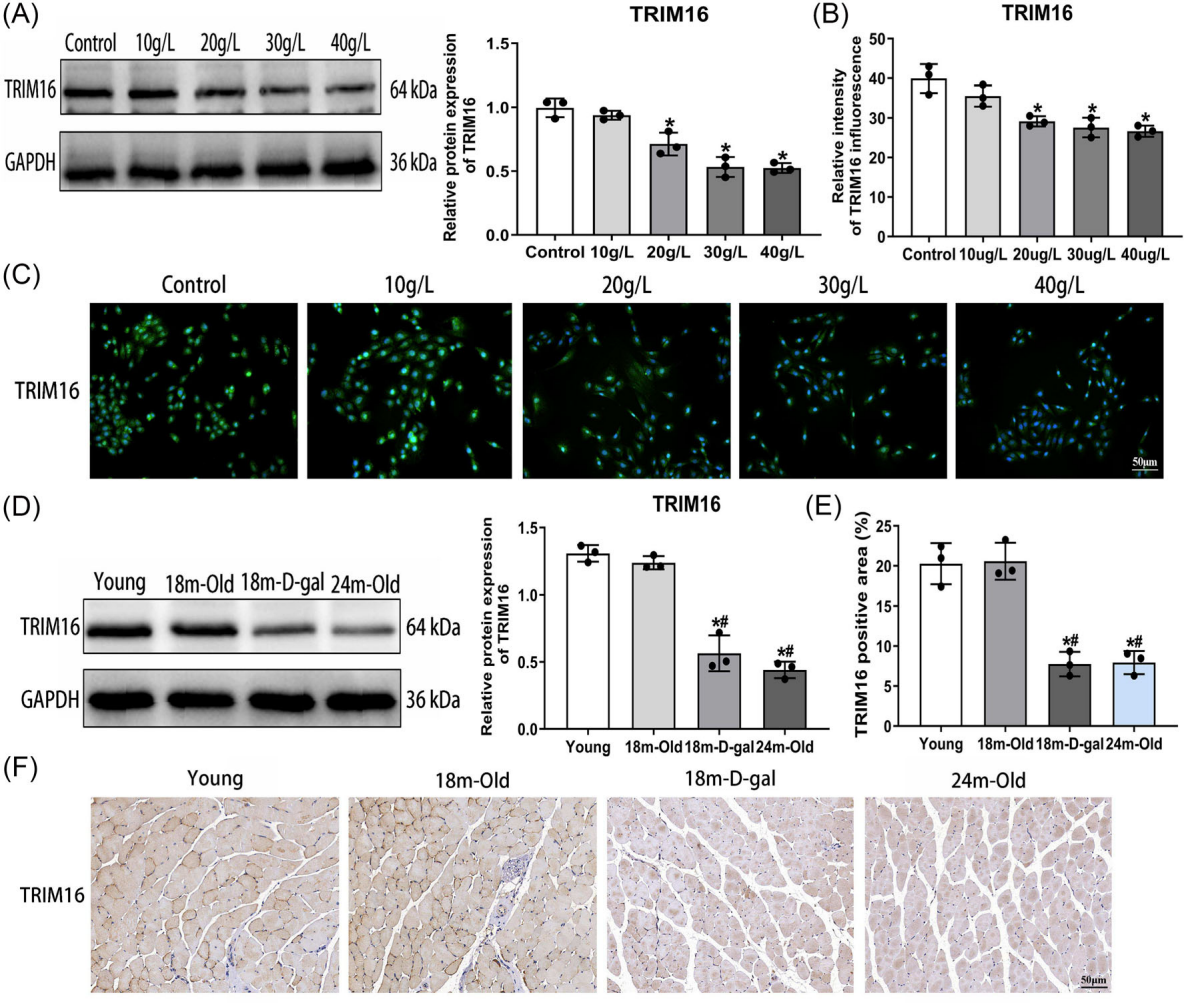
Downregulation of TRIM16 in skeletal muscle of aged mice and D‐galactose‐induced senescent muscle cells. (A) Western blot analysis of TRIM16 protein expression in D‐gal‐induced senescent muscle cells. (B, C) Immunofluorescence assessment of TRIM16 expression in D‐gal‐induced senescent muscle cells (scale bar = 50 μm). (D) Western blot analysis of TRIM16 protein expression in skeletal muscle. (E, F) Immunohistochemical detection of TRIM16 expression in skeletal muscle (scale bar = 50 μm). All data are presented as mean ± SD, *n* = 3 or 6. *P < 0.05 compared with the control group or young group, ^#^ *P* < 0.05 compared with the 18‐month‐old group.

We assessed TRIM16 expression in the skeletal muscle of aged mice and D‐gal‐induced senescent muscle cells. Both western blotting and immunofluorescent staining showed a significant decrease in TRIM16 expression with increasing D‐gal concentrations (Figure 4A–C). Additionally, western blotting and immunohistochemical staining revealed a marked reduction in TRIM16 expression in the 18‐month‐D‐gal and 24‐month‐old groups compared with the young and 18‐month‐old groups (Figure 4D–F). These findings suggest that TRIM16 plays a crucial role in the aging process of skeletal muscle.

### TRIM16 downregulation induces oxidative stress and myotube atrophy in C2C12 muscle cells

**Figure 5:**
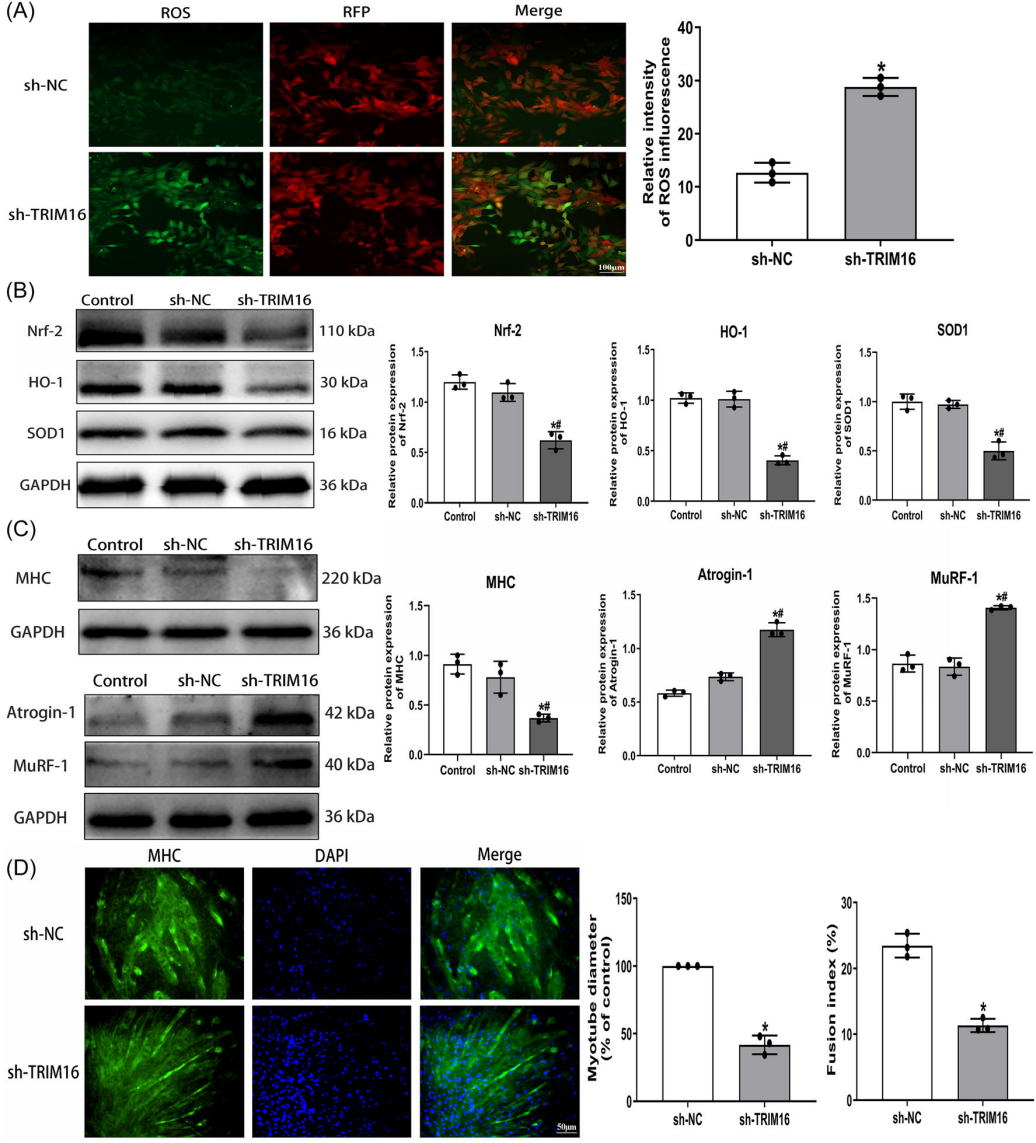
TRIM16 downregulation induces oxidative stress and myotube atrophy in C2C12 muscle cells. (A) Immunofluorescence assessment of ROS levels (scale bar = 100 μm). (B) Western blot analysis revealing protein expression of antioxidant indicators. (C) Western blot detection of myogenic differentiation and muscle atrophy markers protein expression. (D) Immunofluorescence detection of MHC for myotube differentiation (scale bar = 50 μm). All data are presented as mean ± SD, *n* = 3. *P < 0.05 compared with the control or sh‐NC group, ^#^ *P* < 0.05 compared with the sh‐NC group.

We utilized lentiviral transfection to knock down TRIM16 expression in C2C12 myoblasts followed by induction of myoblast differentiation into myotubes. Western blot and immunofluorescence analyses revealed a reduction of over 50% in TRIM16 expression in the TRIM16‐knockdown group (sh‐TRIM16) compared with both the normal control and negative control lentivirus group (sh‐NC) (Figure S4a, b). Subsequently, a significant increase in cytoplasmic ROS levels was noted in the sh‐TRIM16 group compared with the sh‐NC group (Figure 5A). Western blot analysis showed a significant decline in the expression of antioxidant proteins Nrf‐2, HO‐1, and SOD1 in the sh‐TRIM16 group (Figure 5B), accompanied by increased expression of Keap1 and NOX4 (Figure S4c). Moreover, TRIM16 knockdown led to decreased expression of MHC, MyoD, and MyoG, alongside increased expression of Atrogin‐1 and MuRF‐1 (Figures 5C and S4d). Additionally, immunofluorescence staining of MHC revealed significantly smaller myotube dimensions and a reduced fusion index in the sh‐TRIM16 group compared with sh‐NC groups (Figure 5D). These findings indicate that TRIM16 knockdown results in oxidative stress and inhibition of myogenic differentiation leading to myotube atrophy.

### TRIM16 overexpression ameliorates oxidative stress and myotube atrophy in D‐galactose‐treated senescent muscle cells

**Figure 6:**
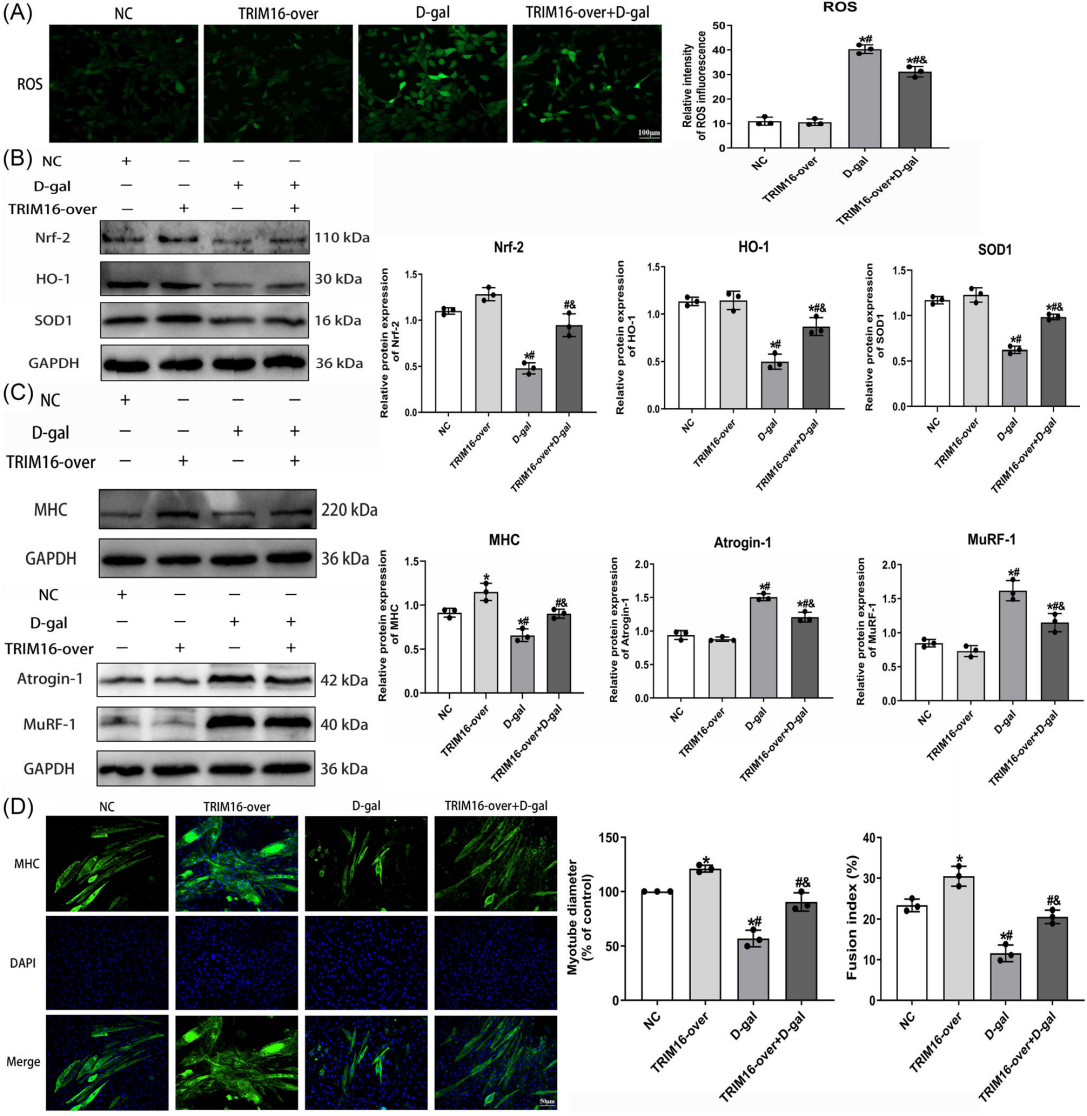
TRIM16 overexpression ameliorates oxidative stress and myotube atrophy in D‐galactose‐treated senescent muscle cells. (A) Immunofluorescence detection of ROS levels (scale bar = 100 μm). (B) Western blot analysis of antioxidant indicators protein expression. (C) Western blot analysis of myogenic differentiation and muscle atrophy markers protein expression. (D) Immunofluorescence detection of MHC for myotube differentiation (scale bar = 50 μm). All data are presented as mean ± SD, *n* = 3. *P < 0.05 compared with the NC group, ^#^ *P* < 0.05 compared with the TRIM16‐over group, ^&^ *P* < 0.05 compared with the D‐gal group.

Both western blotting and immunofluorescence analyses demonstrated a significant increase in TRIM16 expression in the TRIM16‐overexpressing (TRIM16‐over) group compared with the normal control and negative control lentivirus (NC) groups (Figure S5a,b). Assessment of ROS fluorescence revealed that TRIM16 overexpression significantly mitigated the increase in ROS induced by D‐gal (Figure 6A). Furthermore, there was a significant increase in antioxidant protein expression (Figure 6B). Conversely, Keap1 and NOX4 levels decreased, effectively mitigating the inhibitory effect of D‐gal on the antioxidant response (Figure S5c). Additionally, measurement of oxidative stress biomarkers in the cell culture supernatant showed that TRIM16 overexpression attenuated the D‐gal‐induced decrease in SOD activity and GSH levels, while increasing MDA levels (Figure S5d). Western blotting also demonstrated that TRIM16 overexpression countered the inhibitory effect of D‐gal on myogenic differentiation markers and the upregulation of muscle atrophy markers (Figures 6C and S5e). Furthermore, immunofluorescent staining of MHC revealed that TRIM16 overexpression attenuated the reduction in myotube diameter and fusion index induced by D‐gal intervention (Figure 6D).

### TRIM16 overexpression reverses decline in antioxidant activity and muscle atrophy in aged mice

**Figure 7:**
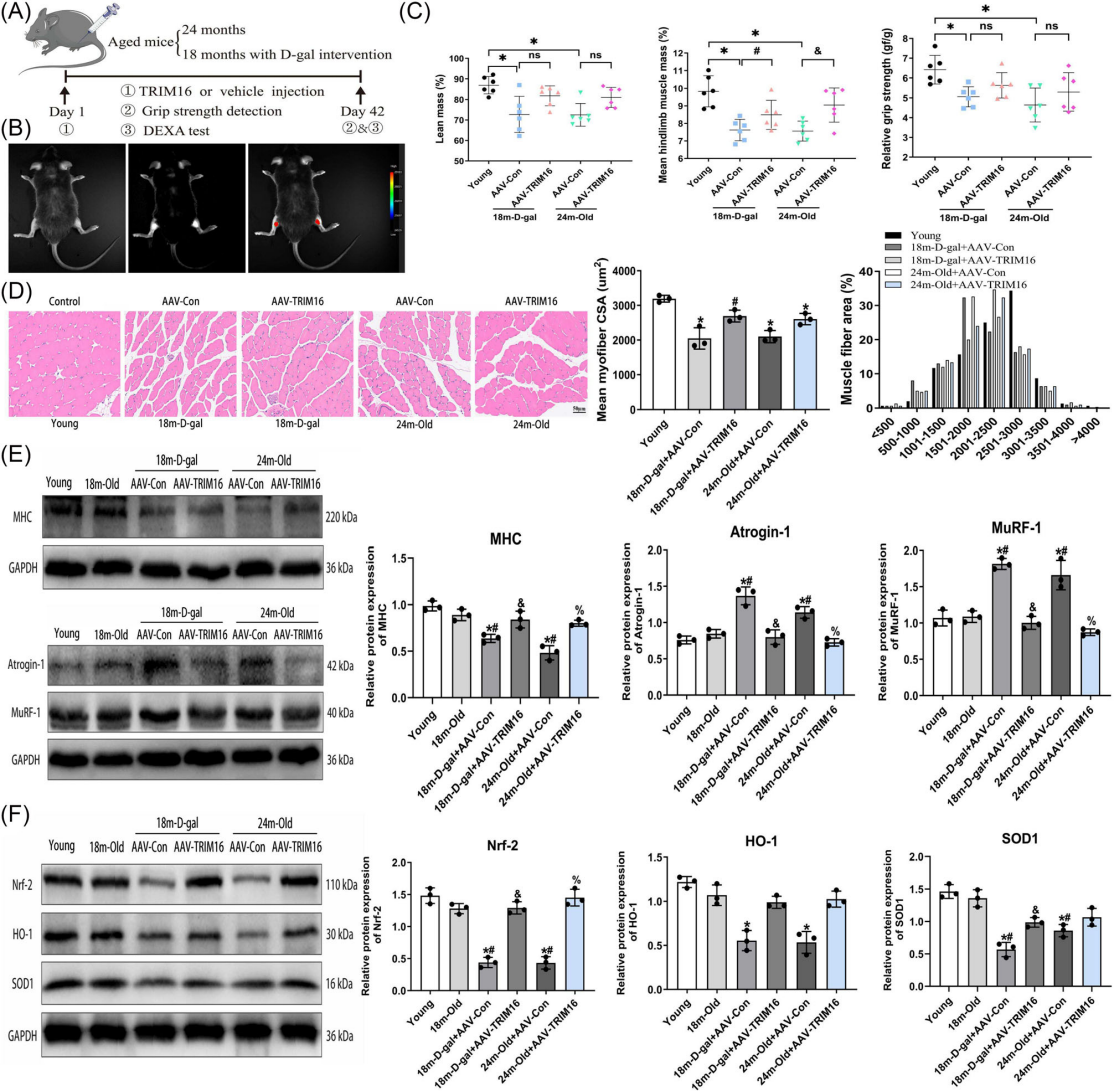
TRIM16 overexpression reverses decline in antioxidant activity and muscle atrophy in aged mice. (A, B) Schematic representation of in‐situ AAV9 injection into skeletal muscle and fluorescent imaging of mice. (C) Assessment of changes in lean mass, mean hindlimb muscle mass, and relative grip strength following in‐situ overexpression of TRIM16 (the Kruskal–Wallis test was employed for the lean mass data sets exhibiting non‐normal distributions, *P*‐value = 0.0053). (D) Morphological observation of muscle fibres through HE staining, with quantitative analysis of the CSA of muscle fibres (scale bar = 50 μm). (E) Western blot analysis depicting the expression of myogenic differentiation and muscle atrophy markers. (F) Western blot analysis illustrating the expression of antioxidant indicators (the Kruskal–Wallis test was employed for the HO‐1 data sets exhibiting non‐normal distributions, *P*‐value = 0.0132). All data are presented as mean ± SD, *n* = 3 or 6. *P < 0.05 compared with the young group, ^#^ *P* < 0.05 compared with the 18‐month‐old group or 18‐month‐D‐gal+AAV‐Con group, ^&^ *P* < 0.05 compared with the 18‐month‐D‐gal+AAV‐Con group or 24‐month‐old+AAV‐Con group, ^%^ *P* < 0.05 compared with the 24‐month‐old+AAV‐Con group.

To explore whether TRIM16 overexpression could protect against age‐related sarcopenia, we administered intramuscular injections of AAV encoding TRIM16 to mice from the 18‐month‐D‐gal and 24‐month‐old groups (Figure 7A). Live mouse fluorescent imaging showed a significant increase in fluorescence intensity in the GAS muscle region of mice injected with TRIM16‐overexpressing AAV (Figure 7B). Additionally, the use of GFP‐tagged AAV confirmed successful in vivo transfection (Figure S6a). However, the injection of the virus into the GAS muscle did not lead to an increased expression of TRIM16 in the heart, liver, kidney, and rectus femoris tissues, indicating that local administration of the virus may have limited influence on other tissues (Figure S6b). DEXA results showed that TRIM16 overexpression notably mitigated the decline in mean hindlimb lean mass in aged mice, although there was no significant increase in lean mass (Figure 7C). Grip strength in the AAV‐TRIM16 groups exhibited an increase compared with the AAV‐Con groups, though this difference lacked statistical significance (Figure 7C).

Additionally, western blot analysis revealed a significant increase in TRIM16 expression in GAS muscle following TRIM16 overexpression (Figure S6c). HE staining showed that TRIM16‐overexpressing mice displayed an increase in the CSA of muscle fibres and a shift in fibre distribution towards the right (Figure 7D). TEM analysis indicated that TRIM16 overexpression improved myofibrillar alignment and the fuzziness of sarcomeres in the GAS muscles of aged mice (Figure S6d). TRIM16 overexpression partially restored the expression of myogenic differentiation and muscle atrophy markers (Figures 7E and S6e). Additionally, it enhanced the downregulation of antioxidant proteins and inhibited the upregulation of Keap1 and NOX4 in skeletal muscle (Figures 7F and S6f), while also alleviating the decrease in SOD activity and GSH levels, and suppressing the increase in MDA levels (Figure S6g).

### The protective role of TRIM16 in skeletal muscle aging: Potential association with SIRT‐1

**Figure 8:**
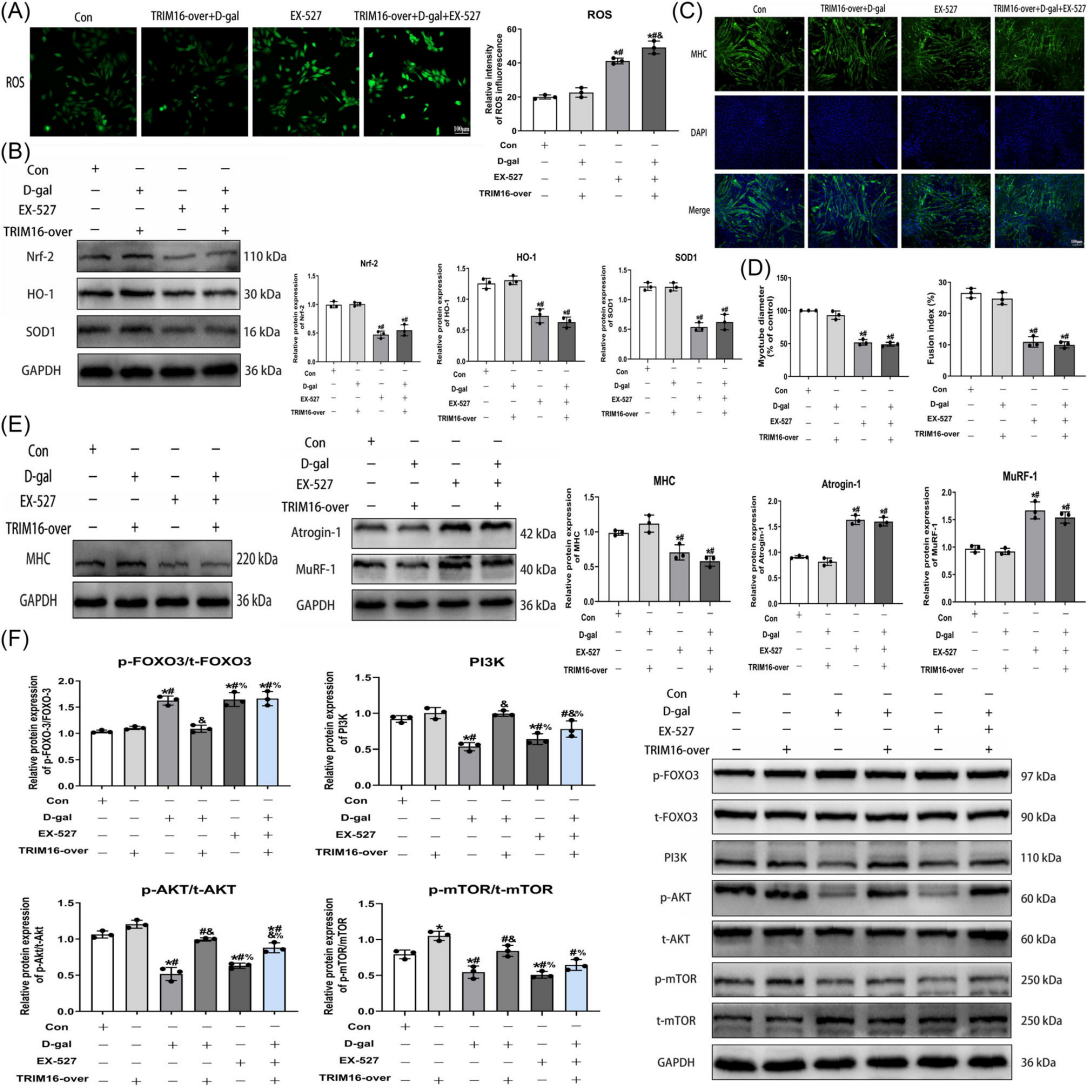
The protective role of TRIM16 in skeletal muscle aging: Potential association with SIRT‐1. (A) Immunofluorescence assessment of ROS levels in myoblasts (scale bar = 100 μm). (B) Western blot analysis of antioxidant markers in myoblasts. (C, D) MHC immunofluorescence examination of myotube differentiation (scale bar = 100 μm). (E) Western blot analysis myogenic differentiation markers and atrophy markers protein expression. (F) Western blot analysis molecules involved in the PI3K/AKT/mTOR pathway and the FOXO3 pathway. All data are presented as mean ± SD, *n* = 3. *P < 0.05 compared with the control group, ^#^ *P* < 0.05 compared with the TRIM16‐over+D‐gal group or TRIM16‐over group, ^&^ *P* < 0.05 compared with the EX‐527 group or D‐gal group, ^%^ *P* < 0.05 compared with the TRIM16‐over+D‐gal group.

Western blot analysis and ROS fluorescence staining showed that inhibiting SIRT‐1 reversed the antioxidant effect attributed to TRIM16, increasing oxidative stress levels (Figures 8A,B and S8a). Additionally, SIRT‐1 inhibition counteracted TRIM16's beneficial impact on myotube atrophy in aging muscle cells (Figures 8C–E and S8b). The regulation of muscle protein synthesis and degradation involves key signalling pathways such as PI3K/AKT/mTOR and FOXO3. TRIM16 overexpression significantly upregulated molecules associated with the PI3K/AKT/mTOR pathway, with no noticeable effect on FOXO3 expression. Furthermore, TRIM16 overexpression partially mitigated FOXO3 pathway activation induced by D‐gal intervention. However, inhibiting SIRT‐1 compromised TRIM16's protective influence on maintaining muscle protein synthesis and degradation balance, with statistical significance (Figure 8F).

## Discussion

The aetiology of sarcopenia in the elderly remains incompletely understood, with age‐associated oxidative stress identified as a crucial factor in its pathogenesis.^26^ Recent investigations have highlighted TRIM16 as a novel regulator of oxidative stress, exerting a protective influence on cells by enhancing the antioxidant system.^11^, ^14^ However, the precise mechanisms by which TRIM16 modulates the antioxidant system in skeletal muscle are not yet fully elucidated. In light of this, we aim to explore the regulatory mechanism of TRIM16 in skeletal muscle, specifically its impact on oxidative stress, in order to offer novel insights into potential therapeutic targets for age‐related sarcopenia.

The D‐gal‐induced cellular aging model, widely recognized, serves as a common experimental technique for simulating aging in vitro. We have developed a protocol to induce cellular aging using D‐gal and established validation procedures. These include quantifying senescent cells, apoptosis rates, assessing cytoskeleton integrity, and measuring aging‐related indicators.^24^, ^27^ Our findings showed that the optimal concentration for inducing cellular aging was 30 g/L of D‐gal. We also noted a simultaneous decrease in TRIM16 expression and an increase in oxidative stress in D‐gal‐induced aging muscle cells. Additionally, these aged cells displayed a potential hindrance to muscle differentiation and an exacerbation of myotube atrophy. Importantly, our results are consistent with prior studies examining the impacts of D‐gal on oxidative stress and muscle atrophy in C2C12 muscle cells.^28^, ^29^

Our findings indicate that reduced TRIM16 expression correlates with decreased levels of key antioxidant molecules, such as Nrf‐2, HO‐1, and SOD1, while the expression of Keap1 and NOX4 increases. This leads to heightened ROS production and subsequent induction of oxidative stress in muscle cells. Nrf‐2, a key regulator in antioxidant stress response, is primarily modulated by the cytoplasmic inhibitor Keap1 and triggers the transcription of antioxidant proteins such as HO‐1 and SOD1.^15^ NOX4, a member of the enzyme family responsible for generating superoxide and hydrogen peroxide, is involved in catalysing ROS production.^30^ The inhibition of antioxidant responses and activation of pro‐oxidant actions lead to ROS accumulation, which in turn upregulates Atrogin‐1 and MuRF‐1 expression.^31^ TRIM16 knockdown in C2C12 muscle cells resulted in decreased MHC, MyoD, and MyoG expression, and increased Atrogin‐1 and MuRF‐1 expression, ultimately leading to myotube atrophy. Furthermore, we observed that the overexpression of TRIM16 partially mitigated the inhibitory effects induced by D‐gal on antioxidant indicators, thereby alleviating oxidative stress and myotube atrophy. These results suggest TRIM16's critical role in regulating the antioxidant system, influencing processes associated with muscle atrophy in C2C12 muscle cells, providing fresh insights into the role of TRIM16 in skeletal muscle aging.

To further explore the regulatory role of TRIM16 in age‐related sarcopenia, we initially established aging mouse models and compared muscle mass and function between aged and young mice. DEXA and grip strength testing revealed a significant reduction in lean body mass and grip strength in aged mice compared with young mice. Structural abnormalities in muscular fibres and decreased CSA were evident in both aging mouse groups. Moreover, suppressed expression of skeletal muscle differentiation markers and upregulated expression of atrophy markers confirmed muscle atrophy in skeletal muscle. These results indicate the successful establishment of the age‐related sarcopenia model. Furthermore, we observed suppressed antioxidant activity and reduced TRIM16 expression in the skeletal muscle of aged mice, increasing the risk of sarcopenia development and progression. In this study, we established a sarcopenia mouse model using two approaches: naturally aged mice reaching 24‐month‐ and 18‐month‐old mice treated with D‐gal via intraperitoneal injection.^23^, ^27^, ^32^ While some studies primarily use 6–8 week young mice for D‐gal intervention, we opted for 18‐month‐old mice to better replicate the characteristics of naturally aged mice.^27^, ^33^ This deliberate choice may have contributed to the lack of significant differences observed between the two sarcopenia mouse models.

Through in situ overexpression of TRIM16 in the skeletal muscle of aged mice, we observed its protective role in age‐related sarcopenia. TRIM16 overexpression partially mitigated muscle atrophy and oxidative stress in aged mice, though complete restoration to normal levels was not achieved. This partial effect may be due to irreversible damage to skeletal muscle function caused by aging or the limited intervention period of TRIM16 overexpression. Current in vivo studies on TRIM16 have mainly investigated the impact of TRIM16 knockdown or overexpression on various tissue organs using systemic and tissue‐specific knockout mice.^14^, ^34^, ^35^ One study investigated TRIM16's role in pathological myocardial hypertrophy by generating cardiac‐specific TRIM16 knockout mice and AAV9‐TRIM16 transfected mice. They found that TRIM16 deficiency significantly worsened myocardial cell hypertrophy, while TRIM16 overexpression had a beneficial effect, reducing myocardial hypertrophy and remodelling through activation of the downstream Nrf‐2 pathway.^34^ Another study showed that TRIM16 overexpression enhances Nrf‐2/ARE antioxidant signalling by downregulating Keap1, protecting neurons from oxygen–glucose deprivation/reoxygenation (OGD/R) injury.^14^ These findings align with our results, suggesting TRIM16's involvement in various conditions such as myocardial hypertrophy, chronic obstructive pulmonary disease, non‐alcoholic fatty liver disease, and periodontal disease.^35^, ^36^, ^37^ Notably, our study is the first to investigate TRIM16's regulatory role in sarcopenia, expanding our understanding of TRIM16 in this context.

SIRT‐1 emerges as a crucial regulator of muscle metabolism, with its activation showing promise as a therapeutic strategy for alleviating muscle atrophy.^38^ Resveratrol, a SIRT‐1 agonist, has demonstrated efficacy in treating muscle atrophy.^22^ Activation of SIRT‐1 enhances FOXO3 phosphorylation and reduces FOXO3 acetylation, inhibiting FOXO3 nuclear translocation and suppressing Atrogin‐1 and MuRF‐1 expression.^39^ Our initial findings suggest that SIRT‐1 expression is influenced in skeletal muscle cells by TRIM16 knockdown or overexpression. Additionally, SIRT‐1 expression is decreased in the skeletal muscle of aged mice, consistent with previous research.^40^ TRIM16 overexpression partly alleviated the decline in SIRT‐1 expression in the skeletal muscle of aged mice (see Data S1). These findings imply that TRIM16 might promote SIRT‐1 activation in skeletal muscle. To further understand TRIM16's protective role through SIRT‐1, we used the SIRT‐1 inhibitor EX‐527, which reversed the positive effects of TRIM16 on oxidative stress and myotube atrophy in senescent muscle cells. This suggests that TRIM16 may indeed play a protective role in aging skeletal muscle through SIRT‐1 activation.

Maintaining the delicate balance between muscle protein synthesis and degradation is crucial for preserving skeletal muscle function. However, aging can disrupt this equilibrium, leading to decreased protein synthesis and increased degradation, ultimately contributing to the onset of sarcopenia.^41^ The PI3K/AKT/mTOR pathway is vital for muscle protein synthesis, while the FOXO3 pathway mediates protein degradation.^42^ We found that TRIM16 stimulates PI3K/AKT/mTOR expression through SIRT‐1 while simultaneously suppressing FOXO3 activation, thus reducing muscle atrophy. Furthermore, our preliminary investigations into the TRIM16‐SIRT‐1 interaction yielded intriguing results. Immunofluorescence staining and co‐immunoprecipitation (CO‐IP) results (see Data S1) revealed an interaction between TRIM16 and SIRT‐1. However, these findings are preliminary, leaving several questions unanswered. It is uncertain whether the absence of the TRIM16‐SIRT‐1 complex would hinder TRIM16's antioxidant function in skeletal muscle, and whether the SIRT‐1 inhibitor EX‐527 disrupts this complex. Hence, further investigations are needed to clarify the specific mechanisms underlying their interaction.

Our research reveals that TRIM16 activation stimulates antioxidant responses and mitigates muscle atrophy through SIRT1, offering protection against skeletal muscle aging. This novel insight suggests that TRIM16 could be a potentially valuable target for preventing and treating age‐related sarcopenia.

## Funding

This work was financially supported by funding from the National Natural Science Foundation of China (No. 82301769) and the Postdoctoral Science Foundation of Chongqing Science and Technology Bureau (No. CSTB2022NSCQ‐BHX0005).

## Conflict of interest

All authors declare that they have no conflicts of interest.

## Supporting information


**Data S1.** Supporting Information
