# Supplementary material for: TRIM16 facilitates SIRT‐1‐dependent regulation of antioxidant response to alleviate age‐related sarcopenia
Source: J Cachexia Sarcopenia Muscle. 2024 Aug 27;15(5):2056–70. doi: 10.1002/jcsm.13553 (PMC11446700; doi:10.1002/jcsm.13553)

Supplementary Material

1. Materials and methods

Table 1 Sequence of synthesized shRNA targeting TRIM16 and control scrambled shRNA

Gene name	Top strand	Bottom strand
shRNA targeting TRIM16	GATCCGCTTCTCCTGGAGC ATCCATTGGAACTCGAGTT CCAATGGATGCTCCAGGAG AAGCTTTTTTG	AATTCAAAAAAGCTTCTC CTGGAGCATCCATTGGAA CTCGAGTTCCAATGGATGC TCCAGGAGAAGCG
Control scrambled shRNA	GATCCGTTCTCCGAACGTG TCACGTAATTCAAGAGATT ACGTGACACGTTCGGAGAA TTTTTTC	AATTGAAAAAATTCTCCG AACGTGTCACGTAATCTCT TGAATTACGTGACACGTTC GGAGAACG

Table 2 Primary antibodies were utilized for Western blotting, immunofluorescence, and immunohistochemistry

Antibody	Company (Lot.)	Working dilutions
TRIM16	Affinity Biosciences, AF0352	WB: 1/1000 IHC: 1/100
TRIM16	Santa Cruz, sc-398851	IF: 1/100 CO-IP: 1-2ug
MHC	R&D, MAB4470	WB: 1/1000 IF: 1/200
SIRT-1	Proteintech, 13161-1-AP	IF: 1/200 CO-IP: 0.5-4ug
SIRT-1	Cell Signaling Technology, #8469	WB: 1/1000
Desmin	Proteintech, 16520-1-AP	IF: 1/200
Laminin	Abcam, ab11575	IF: 1/200
P53	Proteintech, 10442-1-AP	WB: 1/1000

P21	Proteintech, 28248-1-AP	WB: 1/1000
P16	Zenbio, R23897	WB: 1/500
MyoD	Proteintech, 18943-1-AP	WB: 1/1000
MyoG	Zenbio, 382257	WB: 1/500
Atrogin-1	Zenbio, R383217	WB: 1/1000
MuRF-1	Proteintech, 55456-1-AP	WB: 1/1000
Nrf-2	Proteintech, 80593-1-RR	WB: 1/1000
HO-1	Zenbio, R24541	WB: 1/500
SOD1	Zenbio, R25829	WB: 1/500
Keap1	Proteintech, 10503-2-AP	WB: 1/1000
NOX4	Proteintech, 14347-1-AP	WB: 1/1000
FOXO3	Zenbio, 381451	WB: 1/1000
p-FOXO3(Ser235)	Zenbio, 381118	WB: 1/1000
AKT	Proteintech, 10176-2-AP	WB: 1/2000
p-AKT(Ser473)	Proteintech, 28731-1-AP	WB: 1/2000
PI3K	Affinity Biosciences, AF5112	WB: 1/1000
m-TOR	Affinity Biosciences, AF6308	WB: 1/1000
p-mTOR(Ser2448)	Affinity Biosciences, AF3308	WB: 1/1000
GAPDH	Proteintech, 10494-1-AP	WB: 1/4000

Cell viability

Cells were seeded in 96-well plates at a density of 2×10^3 cells per well and treated with various concentrations of D-gal or EX-527 for 24 and 48 hours. Following treatment, cell viability was assessed using the Cell Counting Kit-8 (CCK-8) reagent (Dojindo, Japan) according to the manufacturer's instructions. The absorbance at 450 nm was measured using a microplate reader to quantify cell viability.

Mitochondrial membrane potential measurement

The mitochondrial membrane potential in C2C12 cells was evaluated using the JC-1 Assay Kit (Beyotime Biotechnology, China). Cells cultured in confocal dishes were treated with 100 μl of JC-1 staining solution (5μg/mL) at 37°C for 20 minutes in the dark. After washing with ice-cold JC-1 buffer, the cell nuclei were stained with Hoechst 33342 Staining Solution (Beyotime Biotechnology, China). Images were captured using laser confocal scanning microscopy, and the mitochondrial membrane potential was analyzed based on the relative fluorescence intensity from mitochondrial JC-1 monomers or aggregates.

Co-immunoprecipitation

Lysed C2C12 cells were harvested by centrifugation at 12000 rpm for 15 minutes at 4°C, and the supernatant was collected. The supernatant was then incubated overnight at 4°C with either TRIM16 or SIRT-1 antibody. Subsequently, 30 μL of protein A/G magnetic beads (MedChemExpress, MCE, USA) were added, and the mixture was incubated for 2-4 hours at 4°C. Samples were then separated using a magnet separator, and the supernatant was discarded. Following three washes with lysis buffer, the samples were supplemented with protein loading buffer. After heating at 100°C for 10 minutes, immunoprecipitated proteins were eluted from the magnetic beads. Finally, the samples were immunoblotted with antibodies against SIRT-1 or TRIM16.

2. Results

TRIM16 implicates SIRT-1 expression regulation in skeletal muscle

The results showed that SIRT1 expression decreased in C2C12 cells with TRIM16 knockdown, while TRIM16 overexpression significantly increased SIRT1 expression (Figure S7a). To further investigate the protective role of TRIM16 through SIRT-1 in skeletal muscle aging, the selective SIRT1 inhibitor, EX-527, was utilized. The CCK8 assay demonstrated a significant decrease in cell proliferation activity at 24 and 48 hours with increasing concentrations of EX-527 (Figure S7b). Western blotting confirmed that EX-527 significantly suppressed SIRT-1 expression at concentrations of 100 uM and 125 uM (Figure S7c). Subsequently, 100 uM was chosen as the intervention concentration of EX-527 to ensure functional activity of cells in subsequent experiments. Furthermore, TRIM16 overexpression reversed the D-gal-induced decrease in SIRT-1 expression; however, this positive impact was blocked by the use of EX-527 inhibitors (Figure S7d). Additionally, SIRT-1 expression in the skeletal muscle of aged mice was significantly lower compared to young control mice, and TRIM16 overexpression could alleviate SIRT-1 downregulation in skeletal muscle (Figure S7e,f).

The protective role of TRIM16 in skeletal muscle aging: Potential association with SIRT-1

We conducted preliminary investigations into the interaction between TRIM16 and SIRT-1. Our immunofluorescence results revealed predominant expression of TRIM16 and SIRT-1 in the myoblast nucleus (Figure S8c). Subsequently, we utilized CO-IP to explore the potential interaction between TRIM16 and SIRT-1. In the first gel, anti-TRIM16 was utilized for immunoprecipitating SIRT-1. Both the TRIM-16 (64 kDa) and SIRT-1 (110 kDa) protein bands were observed in both the Input (positive control) group and the IP group. Moving to the second gel, we employed anti-SIRT-1 to immunoprecipitate TRIM16 protein, revealing the presence of both the SIRT-1 (110 kDa) and TRIM16 (64 kDa) protein bands in both the Input and IP

groups. Importantly, no bands were detected in the IgG group. These results strongly indicate a potential interaction between TRIM16 and SIRT-1 proteins (Figure S8d).

3. Supplementary figures

Figure S1 D-galactose-induced senescence in C2C12 muscle cells. (a) Cell proliferation activity assessed by CCK-8 assay. (b) Immunofluorescence staining of C2C12 cytoskeleton using Desmin (scale bar = 50 μm). (c) Staining of mitochondrial membrane potential using JC-1 (scale bar = 50 μm). All data are presented as mean ± SD, n = 3. *P < 0.05 compared with the control group.

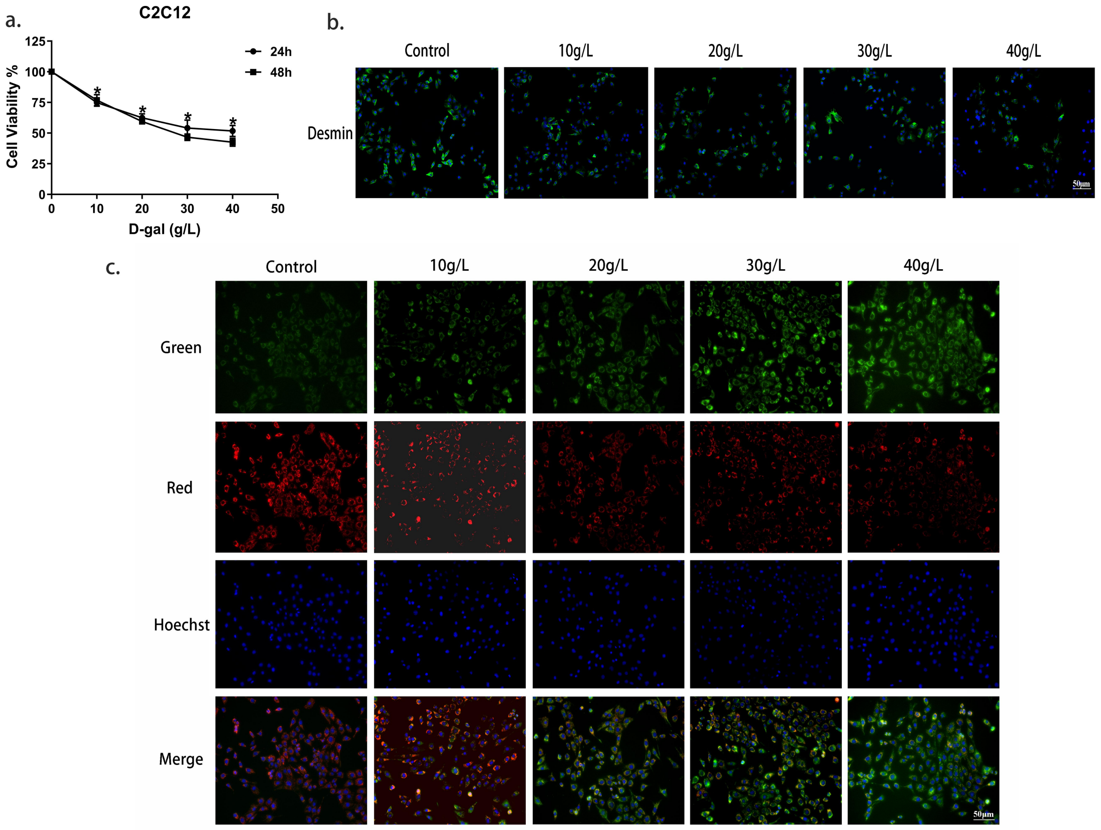

Figure S2 Oxidative stress and myotube atrophy in D-galactose-induced senescence of C2C12 muscle cells. (a) Western blot analysis of protein expression related to oxidative stress indicators. (b) Western blot analysis of protein expression pertaining to muscle differentiation markers. All data are presented as mean ± SD, n = 3. *P < 0.05 compared with the control group.

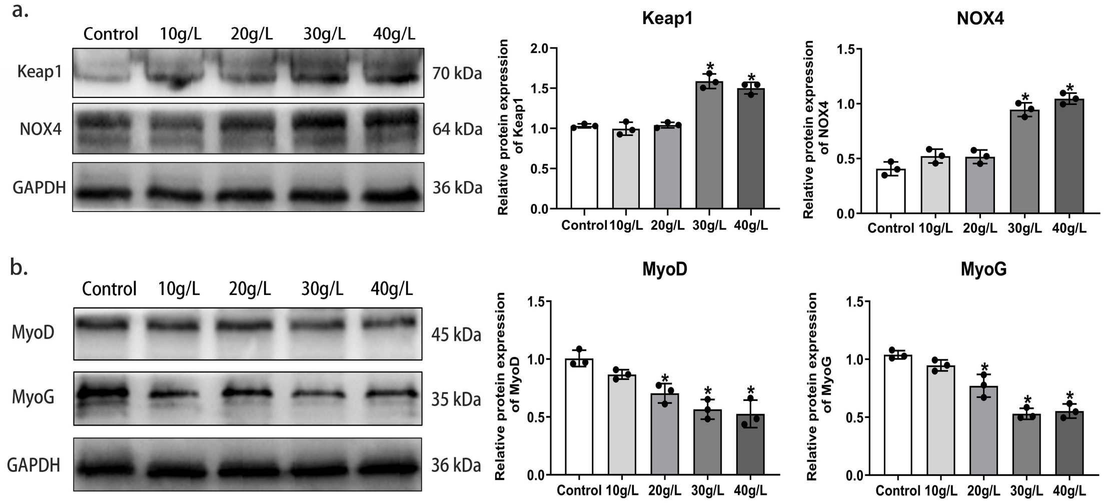

Figure S3 Skeletal muscle atrophy and suppressed antioxidant activity in aged mice. (a) Comparison of body weight among different groups of mice. (b) Fluorescence staining using Laminin to observe the morphology of muscle fibers (scale bar = 50 μm). (c) Western blot analysis of protein expression related to muscle differentiation markers. (d) Western blot analysis of protein expression related to oxidative stress indicators (the Kruskal-Wallis test was employed for the NOX4 data sets exhibiting non-normal distributions, P-value = 0.0020). All data are presented as mean ± SD, n = 6. *P < 0.05 compared with the young group, #P < 0.05 compared with the 18-month-old group.

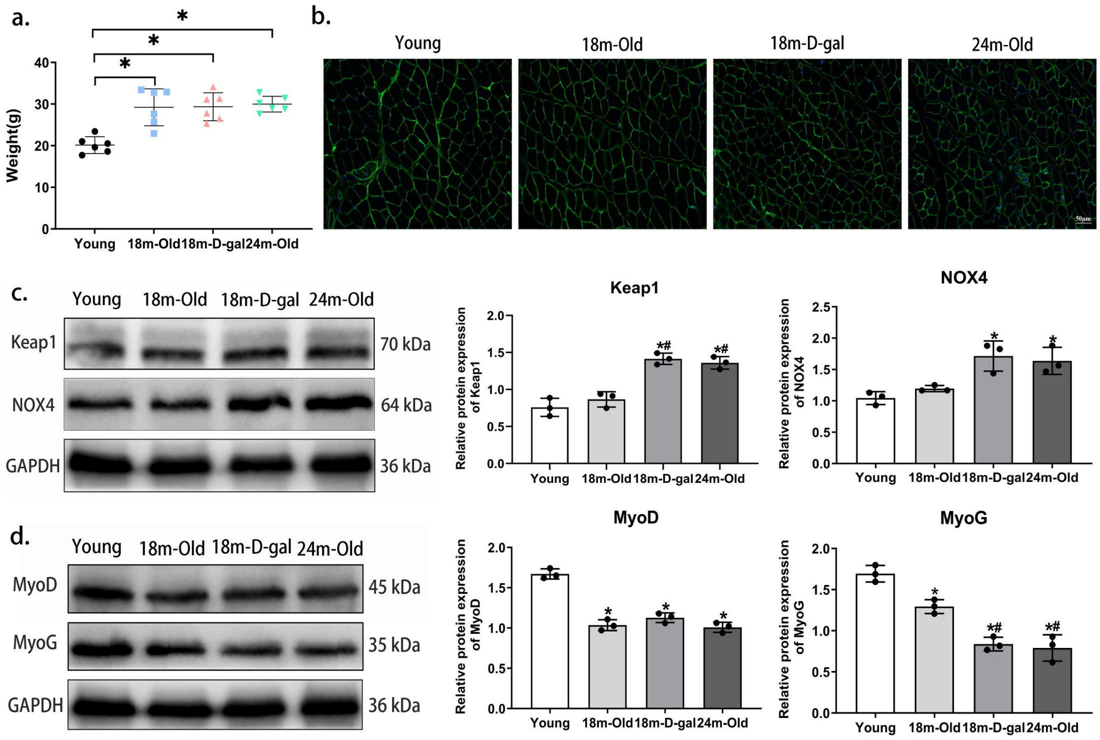

Figure S4 TRIM16 downregulation induces oxidative stress and myotube atrophy in C2C12 muscle cells. (a) Western blot analysis of TRIM16 protein expression. (b) Immunofluorescence detection of TRIM16 expression (scale bar = 50 μm). (c) Western blot analysis of protein expression related to oxidative stress indicators.(d) Western blot analysis of protein expression related to muscle differentiation markers. All data are presented as mean ± SD, n = 3. *P < 0.05 compared with the control or sh-NC group, #P < 0.05 compared with the sh-NC group.

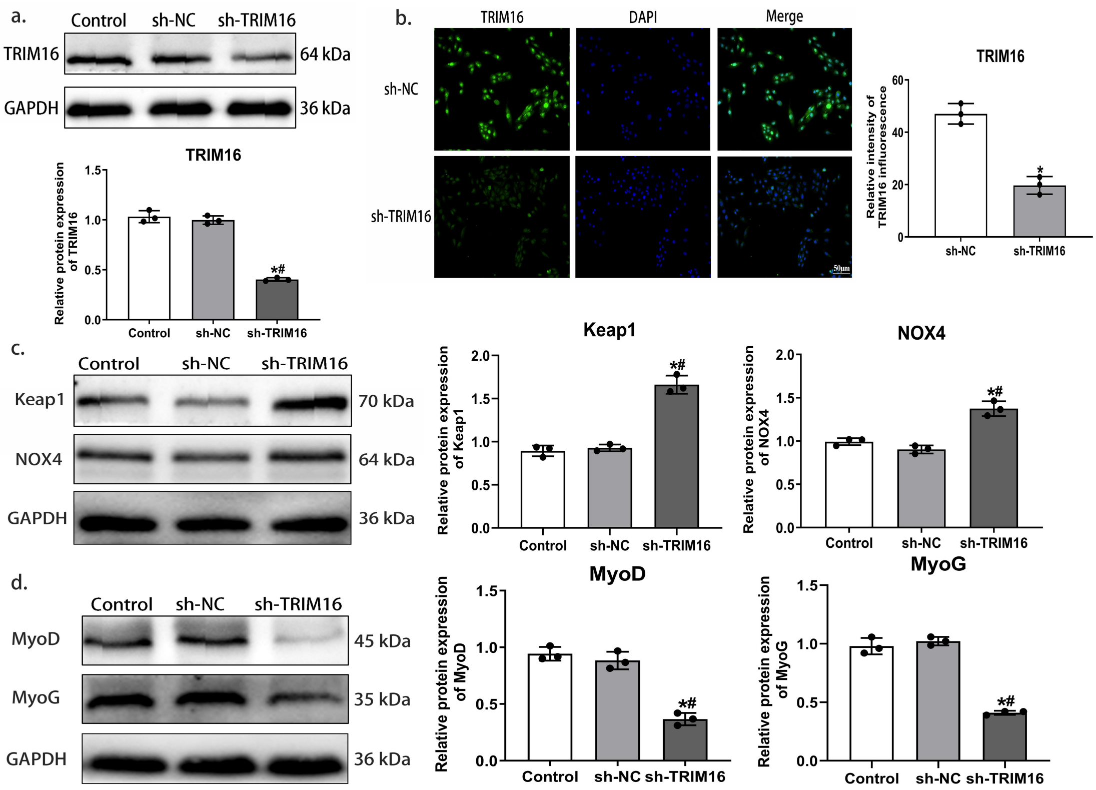

Figure S5 TRIM16 overexpression ameliorates oxidative stress and myotube atrophy in D-galactose-treated senescent muscle cells. (a) Western blot analysis of TRIM16 protein expression. (b) Immunofluorescence detection of TRIM16 expression (scale bar = 50 μm). (c) Western blot analysis of protein expression related to oxidative stress indicators. (d) Measurement of activity expression of oxidative stress indicators GSH, SOD, and MDA in cells. (e) Western blot analysis of protein expression related to muscle differentiation markers. All data are presented as mean ± SD, n = 3. *P < 0.05 compared with the control or NC group, $^{\#}$P < 0.05 compared with the NC group or TRIM16-over group, $^{\&}$P < 0.05 compared with the D-gal group.

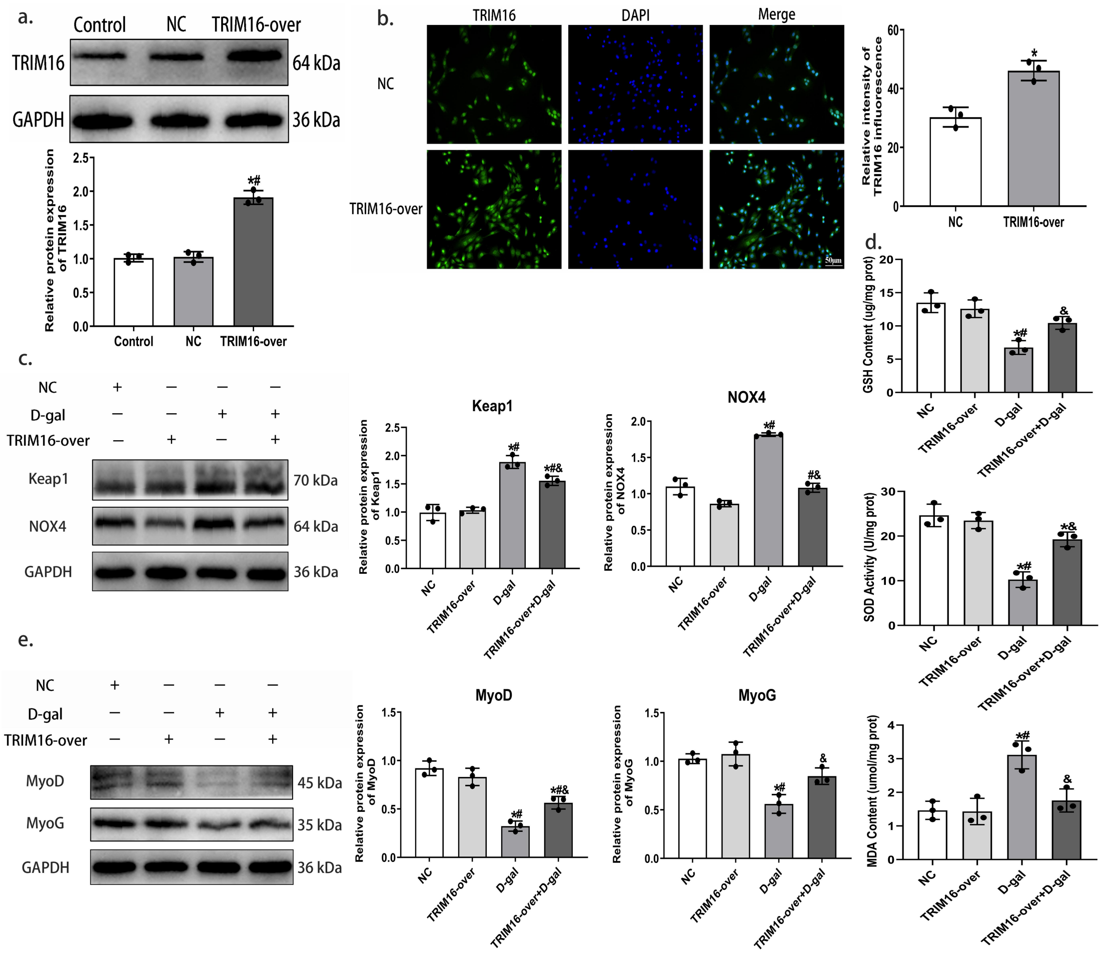

Figure S6 TRIM16 overexpression reverses decline in antioxidant activity and muscle atrophy in aged mice. (a) Detection of AAV9 virus autoluminescence (scale bar = 50 μm). (b) Western blot analysis of TRIM16 protein expression in heart, liver, kidney, and rectus femoris. (c) Assessment of changes in TRIM16 expression. (d) TEM observation of muscle fiber structure (scale bar =1 μm). (e) Western blot analysis of protein expression related to muscle differentiation markers. (f) Western blot analysis of protein expression related to oxidative stress indicators. (g) Measurement of GSH, SOD, and MDA indicator levels in each group. All data are presented as mean ± SD, n = 3. *$P < 0.05$ compared with the young group, $^{\#}P<0.05$ compared with the 18-month-old group or 18-month-D-gal+AAV-Con group, $^{\&}P<0.05$ compared with the 18-month-D-gal+AAV-Con group or 24-month-old+AAV-Con group, $^{\%}P < 0.05$ compared with the 24-month-old+AAV-Con group.

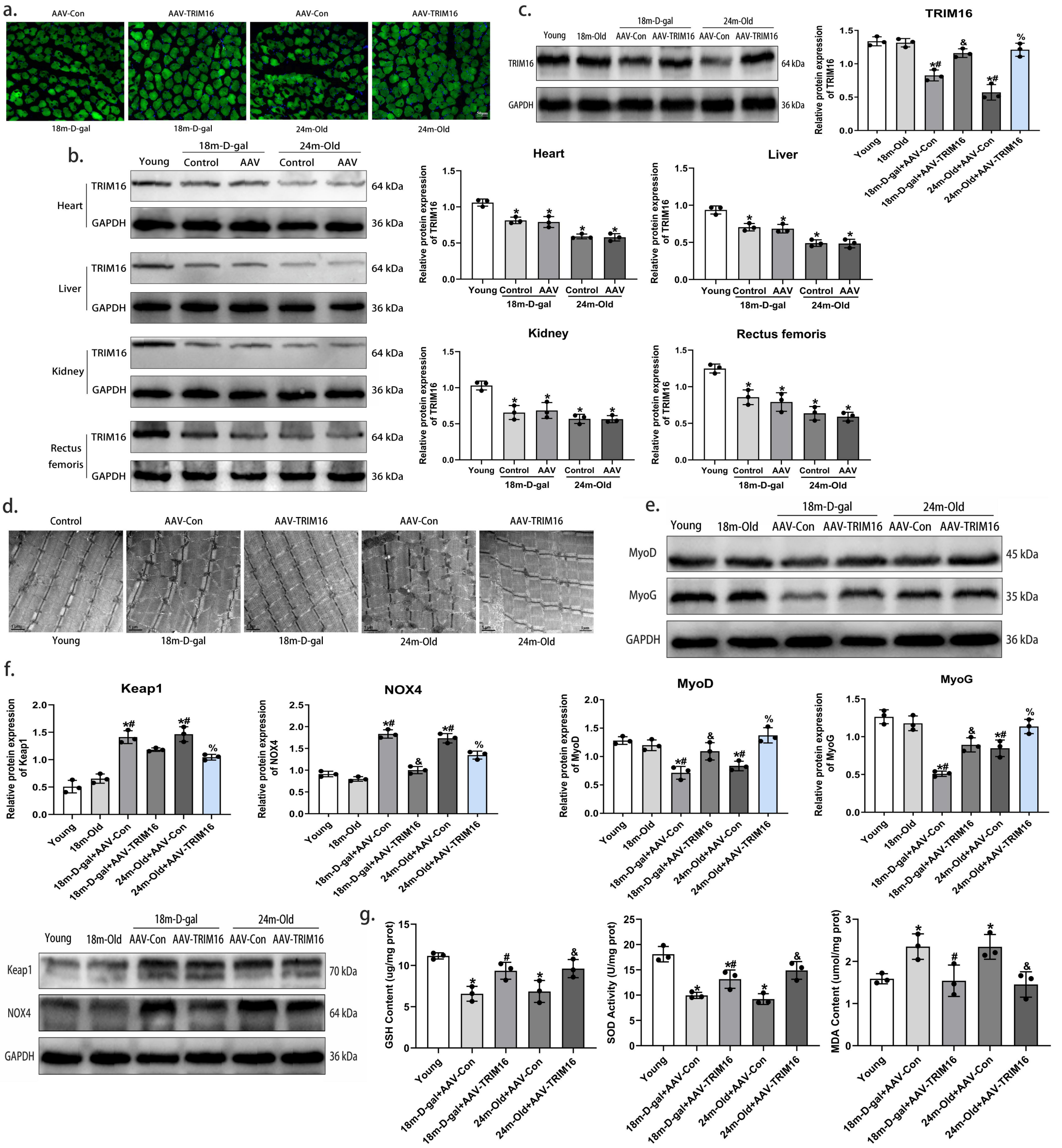
a.
AAV-Con
AAV-TRIM16
AAV-Con
AAV-TRIM16
18m-D-gal
18m-D-gal
24m-Old
24m-Old
b.
Young
18m-D-gal
24m-Old
Control
AAV
Control
AAV
Heart
Liver
Kidney
Rectus femoris
TRIM16
GAPDH
64 kDa
36 kDa
Relative protein expression of TRIM16
c.
TRIM16
Young
18m-Old
18m-D-gal
24m-Old
AAV-Con
AAV-TRIM16
GAPDH
18m-D-gal+AAV-Con
18m-D-gal+AAV-TRIM16
24m-Old+AAV-Con
24m-Old+AAV-TRIM16
d.
Control
AAV-Con
AAV-TRIM16
Young
18m-D-gal
24m-Old
e.
MyoD
MyoG
GAPDH
45 kDa
35 kDa
36 kDa
f.
Keap1
NOX4
MyoD
MyoG
Relative protein expression of Keap1
Relative protein expression of NOX4
Relative protein expression of MyoD
Relative protein expression of MyoG
70 kDa
64 kDa
g.
GSH Content (ug/mg prot)
SOD Activity (U/mg prot)
MDA Content (umol/mg prot)

Figure S7 TRIM16 implicates SIRT-1 expression regulation in skeletal muscle. (a) Western blot analysis of the impact of TRIM16 silencing and overexpression on SIRT-1 protein expression. (b) CCK8 assay to assess the effect of different concentrations of EX527 on myoblast proliferation activity. (c) Western blot analysis of the effect of EX527 on SIRT-1 protein expression in C2C12 muscle cells. (d) Western blot analysis demonstrating the inhibitory effect of EX-527 on the TRIM16 overexpression-induced promotion of SIRT-1 expression. (e,f) Western blot analysis of SIRT-1 protein expression in skeletal muscle of mice. All data are presented as mean ± SD, n = 3. *$P < 0.05$ compared with the control group or 0uM or young group, $^{\#}P< 0.05$ compared with the sh-NC group or NC group or TRIM16-over group or 18-month-old group, $^{\&}P < 0.05$ compared with the D-gal group or 18-month-D-gal+AAV-Con, $^{\%}P < 0.05$ compared with the TRIM16-over+D-gal group or 24-month-Old+AAV-Con.

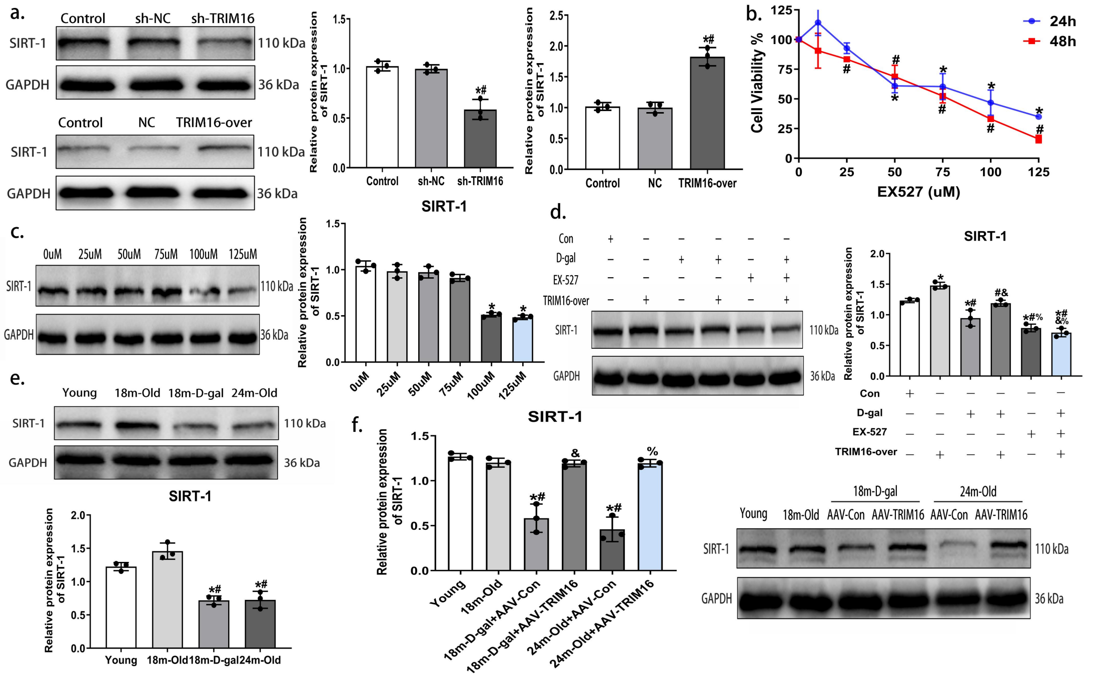

Figure S8 The protective role of TRIM16 in skeletal muscle aging: potential association with SIRT-1. (a) Western blot analysis of protein expression related to oxidative stress indicators. (b) Western blot analysis of protein expression related to muscle differentiation markers. (c) Immunofluorescent staining to observe the localization of TRIM16 and SIRT-1 (scale bar = 50 μm). (d) Co-immunoprecipitation to investigate the interaction between TRIM16 and SIRT-1. All data are presented as mean ± SD, n = 3. *P < 0.05 compared with the control group, $^{\#}$P< 0.05 compared with the TRIM16-over+D-gal group, $^{\&}$P < 0.05 compared with the EX-527 group.

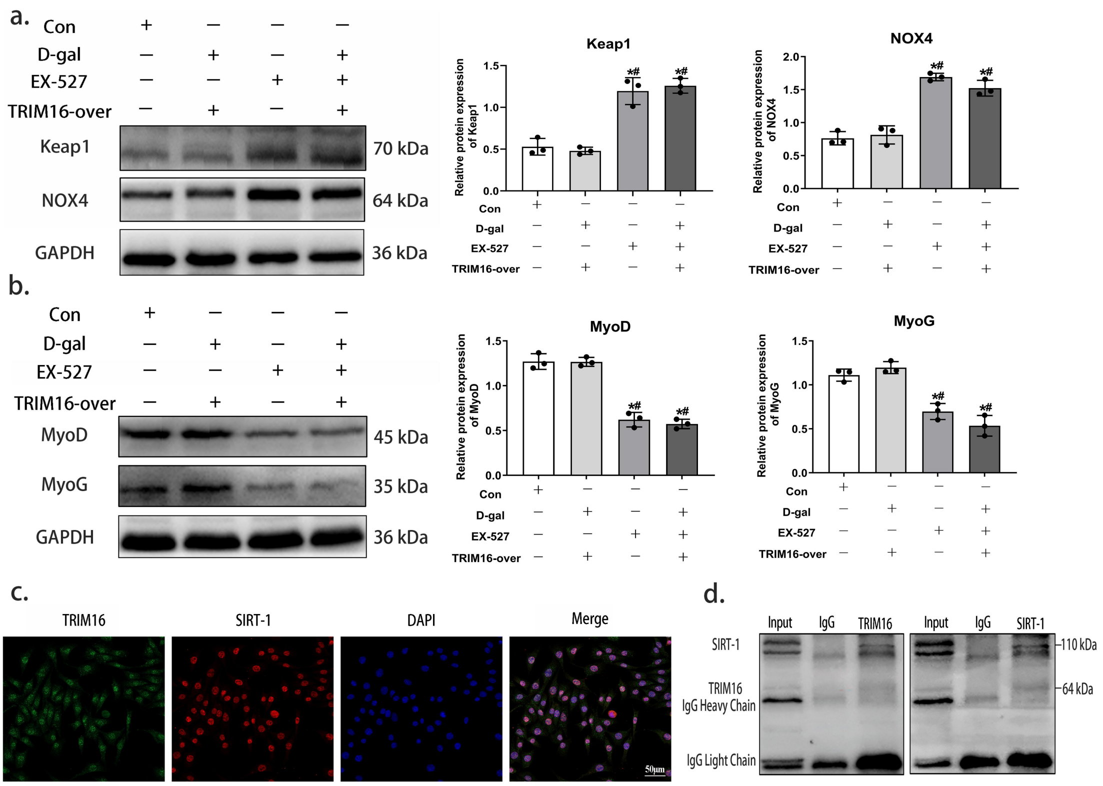

Supplement: Supplementary file 1 — Data S1. Supporting Information [file JCSM-15-2056-s001.pdf]
